# The chordoid glioma-associated PRKCA D463H mutation is a kinase inactive, gain-of-function allele that induces chondrosarcoma in mice

**DOI:** 10.1126/scisignal.adr0235

**Published:** 2025-11-04

**Authors:** Véronique Calleja, Jack C. Henry, Mathias Cobbaut, Joanne Sewell, Karine Rizzoti, Francesca Houghton, Stefan Boeing, Nneka Anyanwu, Sunita Varsani-Brown, Thomas Snoeks, Alejandro Suárez-Bonnet, Simon L. Priestnall, Neil Q. McDonald, Angus J. M. Cameron, Peter J. Parker

**Affiliations:** 1 The Francis Crick Institute, London NW1 1AT, United Kingdom; 2 Barts Cancer Institute, Queen Mary University of London, London, EC1M 6BQ, United Kingdom; 3 The Royal Veterinary School, Hatfield AL9 7TA, United Kingdom; 4 Birkbeck College, London WC1E 7HX, United Kingdom; 5 King’s College London, London WC2R 2LS, United Kingdom

## Abstract

The penetrant D463H mutation in *PRKCA*, which encodes the kinase PKCα, is a biomarker and driver of chordoid glioma, a type of brain cancer. Here, we found that heterozygous knock-in expression of the D463H mutant in mice provoked the development of chondrosarcomas. The mutant protein kinase was catalytically inactive, but no such oncogenic phenotype was observed for the related inactivating mutation D463N, indicating that the lack of activity per se is not the cause of its oncogenic association. In cultured glioma cells, the behaviour of the D463H mutant closely mirrored that of wild-type PKCα and retained ATP binding, contrary to the related D463N mutant. Mechanistically, PKCα D463H displayed quantitative alterations in its interactome compared to that of the wild-type kinase, with enhanced association with epigenetic regulators. This aligned with transcriptomic changes that resembled an augmented PKCα-induced expression program, with enhanced BRD4-, MYC-, and TGF-β–mediated gene signatures. D463H expression reduced the sensitivity of cells to the bromodomain and extra-terminal (BET) inhibitors JQ1 and AZD5153, indicating the functional importance of these pathways. The findings indicate that D463H is a dominant gain-of-function oncogenic mutant, operating through a non-catalytic allosteric mechanism.

## Introduction

There is a breadth of roles assigned to PKC family members in cancer, with examples of both tumour suppressor and tumour promoter actions for individual isoforms (1–4). The complexities surrounding this family of kinases in part reflects isoform specificity and context, however underlying discrepancies may also reflect preconceptions. An intriguing example of this is the role of PKCα in chordoid glioma. In this circumscribed, low-grade but difficult to access CNS tumour, two groups published the finding of completely penetrant heterozygous clonal mutation in *PRKCA* (5, 6). The clonal somatic mutation repeatedly identified was a transversion (c.1387G>C) that alters the catalytic aspartate of PKCα to a histidine residue (D463H). However, no LOH in the 17q24.2 locus, nor other consistent mutations, deletions, amplifications associated with *PRKCA* and no mutations in other oncogenes or tumour suppressors were reported for these tumours. The same D463H mutation was also documented by other groups in a 16 patient clinical study (7) and in two case studies (8, 9), suggesting the mutation is a specific diagnostic marker of chordoid glioma. Intriguingly, extrapolating from the pioneering work on PKA (10, 11) this *PRKCA* mutation would be an inactivating mutation, hence the parsimonious conclusion would be a loss-of-function and a suppressor role for *PRKCA*. However, the heterozygous occurrence of the D463H mutation in chordoid glioma does not align with a classical suppressor role unless through dominant loss-of-function or haploinsufficiency. Such assumptions would seem nevertheless provocative as there is only one mutation found (D463H mutation) while there are many ways to inactivate PKCα.

Understanding the ‘sense’ of action of this penetrant *PRKCA* mutation and the apparent lack of other driver mutations, would offer vulnerability and opportunity to a non/post-surgical intervention in chordoid glioma. To address this, we used genetic models and comparative biochemistry. Here we show that a global heterozygous knock-in of the D463H mutation of *Prkca* in mice drives chondrosarcoma formation in very young neonates. We determined that the *Prkca* D463H mutation is a non-catalytic gain-of-function oncogenic mutation which drives a transcriptomic program resembling an augmented WT response. However, the catalytic activity of PKCα *per se* is not the trigger of the phenotype as a related knock-in inactivating mutation of the same residue (D463N) has no such effect either in a heterozygous or homozygous setting. The distinct behaviour of the D463H mutant is reflected by mutant specific alterations to the PKCα interactome, linking to transcriptomic changes and demonstration of a functional link to BRD4. A model of action of this unique PKCα oncogenic mutant is presented, impacting our wider appreciation of kinase behaviour, and prescribing a distinctive approach to intervention.

## Results

### The heterozygous expression of *Prkca* D463H mutant in mice has a dominant harmful effect causing hindlimb deformation and neonatal death

**Fig. 1:**
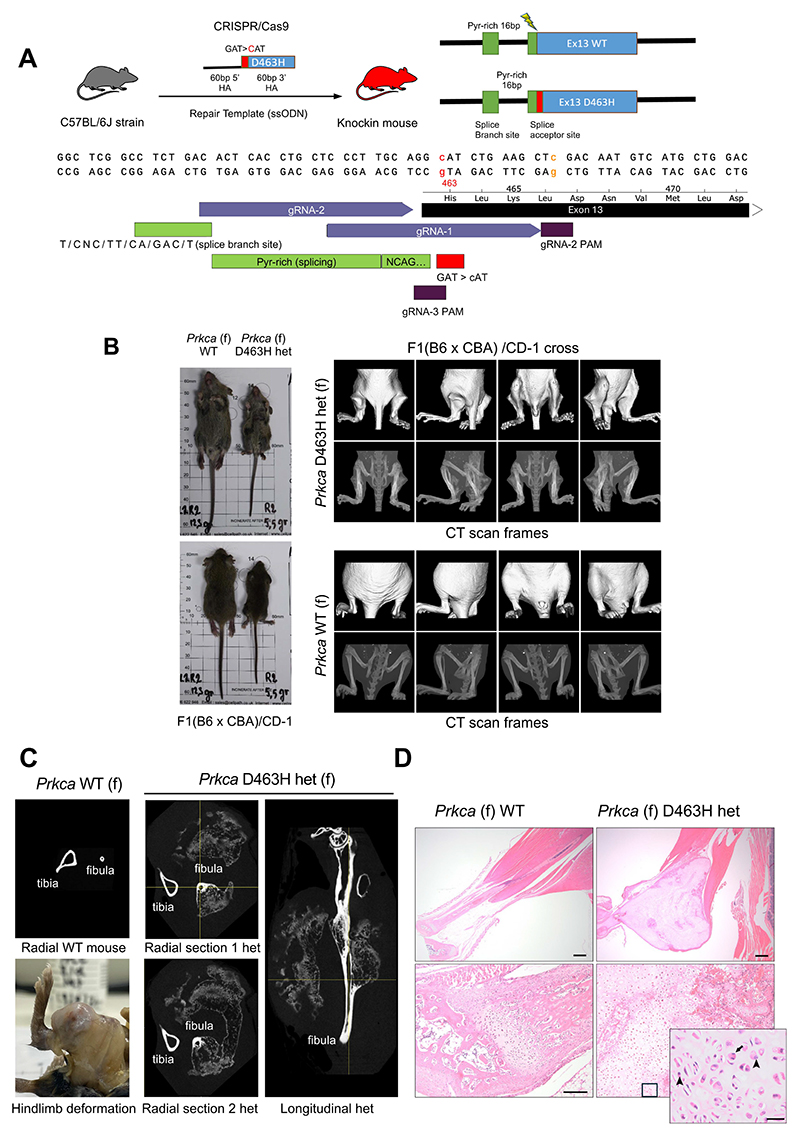
heterozygous knockin mice for PKCα D463H mutation develop chondrosarcoma. (**A**) CRISPR/Cas9 strategy to introduce the D463H point mutation in PKCα in C57BL/6J mice strain. (**B**) Photographs and scans of representative wild-type (WT) and D463H het mutant mice in ventral and dorsal positions. The CT scans focus on the hindlimbs and present multiple views of the bilateral leg deformation of a D463H het mutant mouse (top panels) compared to the WT littermate (bottom panels). (**C**) Representative photograph of the leg of a D463H het mouse and high-resolution CT-scans of radial and longitudinal views of a D463H het mouse compared to a WT littermate. (**D**) H&E stained sections of WT and D463H het mouse hindlimbs at various magnification. Scale bars are 1 mm top, 200 µm bottom. In the zoomed image (scale bar is 20 µm), the arrow is pointing to a mitotic figure and the arrowheads to cells showing cellular atypia. In all panels, 2 animals per genotype were analysed.

To assess the in vivo impact of the PKCα D463H mutation we knocked-in a single allele into C57BL/6J mice using two different guide RNAs (Fig. 1A). From three litters (table S1), 8 pups harboured the mutation (from 11% to 90% penetrance) of which all but 3 had to be culled within 2 weeks due to ill health. Only one male (22% penetrance) survived to breeding age. The WT mice from the same cohorts were healthy, suggesting that the presence of the *Prkca* mutation rather than a side effect of the CRISPR/Cas9 directed knock-in, was responsible for the morbidity of the animals.

In timed matings of the one surviving male mosaic mouse with C57BL/6J females (table S2), 7 heterozygous mice were identified out of 46 pups from 6 different litters, indicating germline transmission and a roughly Mendelian segregation given the mosaic nature of this founder. The *Prkca* D463H heterozygotes pups were smaller or hunchbacked and were malnourished, needing to be culled at about 3 weeks despite the addition of high energy mash nutrition to encourage weight gain.

To determine whether the C57BL/6J mouse background might have contributed to the phenotype, we used outbred CD-1 mice that carry greater genetic diversity, are a more robust strain and produce larger litter sizes than inbred C57BL/6J mice (table S3). Using sperm from the surviving male mosaic mouse, 3 heterozygotes from 4 litters were born. Despite the genetic background change the pups again showed signs of sickness with hindlimb deformation, typically becoming symptomatic at about 2 weeks of age. However, one het male survived longer (11 weeks) and was crossed with CD-1 females producing 4 heterozygotes in 12 progenies (table S4), none of which survived to breeding age, but all of which displayed malnourishment and a hindlimb phenotype (see further below).

The intrinsic constraint on breeding and back-crossing to link genotype to phenotype, led us to perform a second round of gene editing. New mosaic mice were obtained from 8 litters on a C57BL/6J background (4 with guide RNA1 and 4 with guide RNA2) and 8 litters on an F1(B6 x CBA) background (4 with guide RNA1 and 4 with guide RNA2). From these litters 56/103 mice born were mosaic and as observed in the first round of CRISPR knock-ins the mosaic mice but not their WT littermates were malnourished and displayed deformation of the hindlimb. One mosaic male mouse from C57BL/6J background and one male from the F1(B6 x CBA) background that expressed 12% and 18% mutation penetrance respectively were selected for breeding (table S5). Both mice were crossed with two WT CD-1 females producing 15/39 mice from the C57BL/6J male and 6/23 mice from the F1(B6 x CBA) male heterozygous for the D463H mutation. Yet again from the 5 litters obtained (62 mice in total), all the 21 heterozygotes were sickly and displayed a deformation of the hindlimbs. These findings support the conclusion that the heterozygous mutation was a trigger for the malnourished, hindlimb phenotype observed.

### The *Prkca* D463H mutation elicits bilateral hindlimb chondrosarcoma

To address the penetrant hindlimb phenotype of the D463H heterozygotes, representative animals were imaged by computed tomography (CT) (Fig. 1). Whereas the CT scan of the hindlimb showed the extensive loss of weight and the bilateral leg deformation (Fig. 1B), no deformation was visible on the forelimbs (fig. S1A). Furthermore, CT scan of skulls showed a small decrease in size for the heterozygous mice, which was not statistically significant when related to overall body size and weight (fig. S1B).

The longitudinal and radial cross sections of the hindlimbs of representative D463H heterozygotes when compared to a WT littermate clearly showed the aberrant growth of cartilage and bones (Fig. 1C). Effacing the gracilis, semimembranosus, semitendinosus, and gastrocnemius muscles there were bilateral, symmetrical, mesenchymal neoplasms composed of moderately differentiated chondroblasts and chondrocytes surrounded by a pale cartilaginous matrix. There was moderate anisokaryosis and anisocytosis and only a single mitosis was observed in ten high-power fields (400x). The bulk of these neoplasms blended with an outer rim of poorly mineralized woven bone with a population of activated osteoblasts. Occasionally within the neoplasms there were entrapped segments of nerve fibres. There was no evidence of chronic inflammatory changes or haemorrhage. Forelimbs and the remaining soft tissues were histologically unremarkable. Based on both the histological appearance and the infiltrative nature of these proliferative lesions, they were consistent with well-differentiated chondrosarcomas (Fig. 1D). These lesions were associated with morbidity due to muscle and nerve compression and atrophy resulting in the disuse of the hindlimbs. The loss of weight was attributed to the lack of easy access to food due to the competition with the other pups for the mother’s milk. Furthermore, sections from both forelimbs and hindlimbs, lung, heart, liver, kidney, and spleen of WT littermates were also examined and were histologically unremarkable.

Because the PKCα D463H mutation had initially been identified in chordoid glioma tumours originating from β2 tanycytes in the third ventricular region (5, 6) (fig. S1C), we analysed the brains of D463H mutant mice and wild-type controls at the level of the median eminence. Given that mice with the heterozygous D463H mutation did not survive beyond a few weeks of age, the analysis of the adult brain could only be performed on a single mosaic mouse (IRCP 4.1e). This mosaic female (30 weeks, 23% D463H penetrance) was the only surviving littermate of the mosaic founder male IRCP 4.1c (table S1). Additionally, 2-week-old D463H heterozygote and wild-type control mice were analysed (fig. S1, D and E). These analyses did not detect any change in the brain architecture or aberrant growth in any of these animals.

### PKCα D463H mutant is inactive

**Fig. 2:**
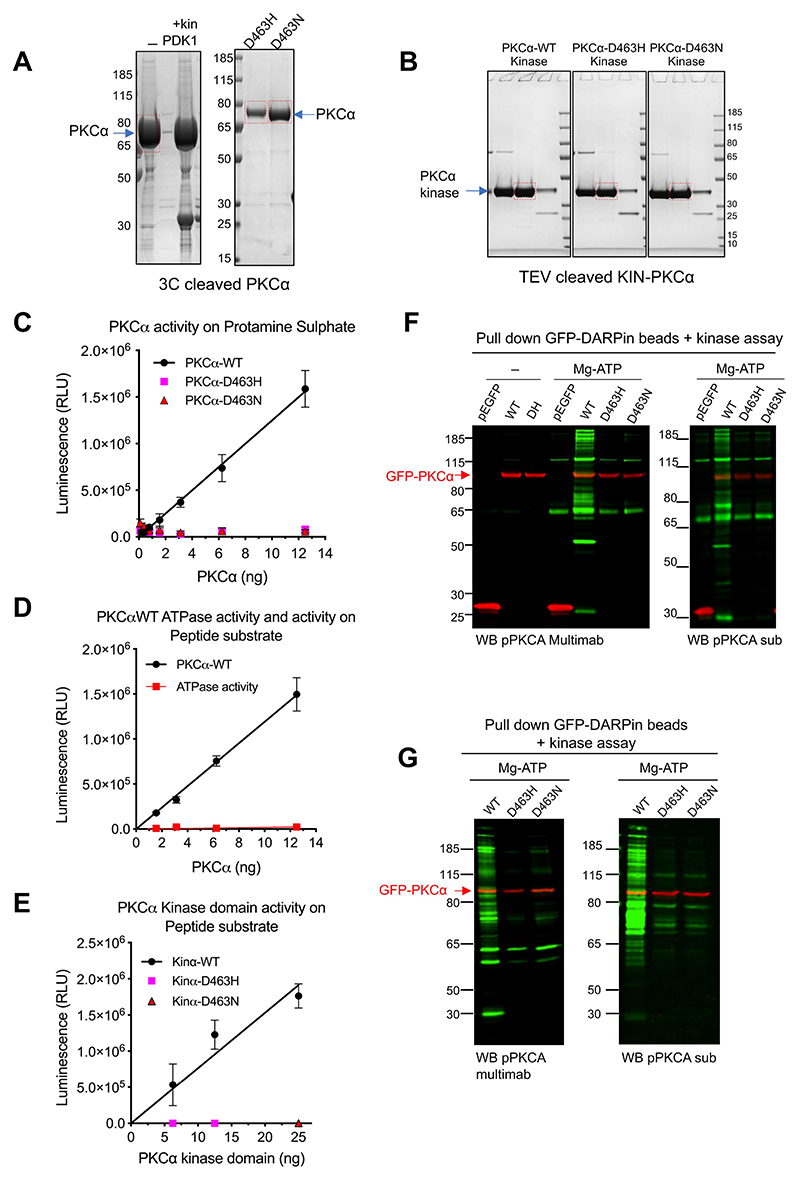
Full-length and kinase-domain PKCα D463H mutants are kinase dead. **(A and B)** Expression of recombinant full-length WT-PKCα and mutants D463H and D463N after C3 cleavage (A) and the kinase domains (KIN) after TEV cleavage of the full-length recombinant PKCα(B) in Sf21 cells. Blots are representative of one of 3 production runs. The fraction used for the experiments is boxed in red. **(C and D)** In vitro kinase activities of recombinant full-length WT or mutant PKCα were measured using the ADP-Glo method on protamine sulphate (C)and on a peptide substrate alongside unspecific ATPase activity (D). Data are means ± SD from *n* = 3 independent experiments. **(E)** In vitro kinase activities of isolated kinase domain activity of recombinant PKCα measured on a peptide substrate. Data are from *n* = 3 independent experiments. **(F)** Representative images of co-precipitation of associated PKCα substrates using DARPin pull down of GFP-tagged WT and mutant PKCα expressed in U87MG cells. Blots are representative of *n* = 5 independent experiments. The substrates ladder is detected by Western blot using two phospho-PKCα substrate-specific antibodies: pPKCA sub and pPKCA multimab in green. PKCα is detected (red) using an antibody to GFP. **(G)** Representative images of a co-precipitation of associated PKCα substrates using DARPin pull down of GFP-tagged WT and mutant PKCα expressed in HCT116 cells and recognised using two phospho-PKCα substrate-specific antibodies: pPKCA sub and pPKCA multimab in green. Blots are representative of *n* = 2 independent experiments

To determine the mechanism by which the PKCα D463H mutant was driving tumour formation, we first analysed the impact of this Asp-to-His change at residue 463 on kinase activity. Recombinant full-length WT and D463H proteins (Fig. 2A and fig. S2) and isolated kinase domains (Fig. 2B) were assessed in vitro for protein kinase catalytic activity on both peptide and protein substrates. As a comparison, the behaviour of a previously characterised mutant at the same site, Asp-to-Asn (D463N), was analysed; this mutant had previously been shown to lack catalytic activity while retaining protein integrity (12). Characteristic activity was observed for the WT protein; however, neither mutant displayed any phosphotransferase activity (Fig. 2, C-E). Assessing ATPase activity showed that this was detectable for the WT protein, albeit orders of magnitude lower than phosphotransferase activity—but again, no activity was observed for either mutant (Fig. 2D). To ensure that there was no compensating function for PKCα derived from mammalian cell expression, we also determined the activities of these proteins on expression in HCT116 (colorectal cancer derived) and U87MG (malignant glioma derived) cells. Using PKCα pull-down/kinase assays on beads, a series of phosphorylated coprecipitated proteins could be detected, but only for WT and not for either of the Asp^463^ mutant proteins (Fig. 2, F and G).

### *Prkca* D463N mutation does not phenocopy *Prkca* D463H in mice

Notwithstanding the lack of an overt tumour driver phenotype in hetero- or homozygous knock-out *Prkca* mice (13), in principle the effect of the D463H inactivation mutation might still be compatible with a dominant-negative impact on a suppressor *Prkca* allele as opposed to a gain-of-function allele. To address this question, we knocked in the *Prkca* D463N inactivating mutation, which is not found in chordoid gliomas but is known to retain protein integrity (12). We used the same guide RNAs as described for the D463H mutation but with a modified repair template to change codon 463 from aspartic acid to an asparagine (D463N). From the 16 C57BL/6J mosaic mice obtained with the D463N mutation (table S6), 3 were bred to assess germline transmission, yielding 15 of 30 pups that were *Prkca* D463N heterozygous from 4 litters (table S7). All mice were asymptomatic and 8 of these offspring were crossed with C57BL/6J, F1(B6 x CBA) or CD-1 mice. In these different backgrounds, a Mendelian ratio of 30 heterozygotes out of 58 pups was observed, and all the 30 heterozygous *Prkca* D463N mice obtained from all backgrounds survived for at least 20 weeks without any health issues (table S8). Hence the monoallelic expression of kinase inactive *Prkca* D463N is not sufficient to drive the chondrosarcoma phenotype.

It remained possible that the D463N mutation might be haploinsufficient failing to “dominate” the WT allele in the manner that the D463H mutation might be argued to do. To address this, we bred the D463N allele (C57BL6 background) to homozygosity (table S9). 12 homozygotes, 14 heterozygotes, and 6 wild-types were generated. None of the mice displayed any abnormal phenotype or organ histology. It is noted that neither mutant appeared to express well in mouse tissues, with the heterozygote D463H+WT expressing 38% that of normal PKCα protein (range 15-65%) and the D463N+WT expressing about 48% (range 41-53); we cannot distinguish WT and mutant proteins and so cannot define their contributions to assess whether D463N versus D463H levels impact phenotype. However, distinctions from ex vivo studies (below) demonstrate distinctive biological properties.

The rapid onset of chondrosarcoma in the *Prkca* D463H heterozygotes, but not in the *Prkca* D463N homozygotes or heterozygotes, indicates that the D463H mutation is a positive driver of neoplastic disease. This suggests that the mutant either has neomorphic properties or that it is a gain-of-function PKCα allele that redefines effector outputs of this protein kinase.

### PKCα D463H behaviour aligns with WT-PKCα properties

With a view to understanding how the D463H mutant varies from the D463N mutant and whether this tracks with WT behaviour or represents neomorphic behaviour, we analysed the effects of the D463H and D463N mutations on biochemical and in cell properties. Thermal shift assays and microscale thermophoresis were conducted to assess whether differences between the mutants might reflect distinct nucleotide binding properties. The mutant proteins showed monophasic melting-temperature (T_m_) curves in a thermal shift assay, unlike WT full-length protein which displayed a biphasic melting-temperature curve. All proteins displayed a shift in melting temperature(s) on binding ATP or ADP (fig. S3A) with some variations in the extent of nucleotide stabilisation (fig. S3B). Despite the biphasic melting behaviour of the WT protein complicating comparisons with the mutants, there is a substantial reduction in ATP affinity for the D463N mutant (shown by higher EC_50_) compared to the WT with the D463H mutant displaying a more modest loss of affinity. Both mutants displayed a similarly reduced affinity for ADP relative to the WT protein (fig. S3C). To address this differential loss of affinity for ATP more directly, we used an orthogonal approach, assessing ATP binding to full-length recombinant PKCα and mutants in vitro with microscale thermophoresis (MST). This revealed that compared to the WT protein, D463H bound to ATP with a ~100-fold lower affinity, while the D462N mutant bound >1000-fold more weakly. These values predict greater ATP occupation of the D463H mutant compared with the D463N mutant at physiological ATP concentrations.

**Fig. 3:**
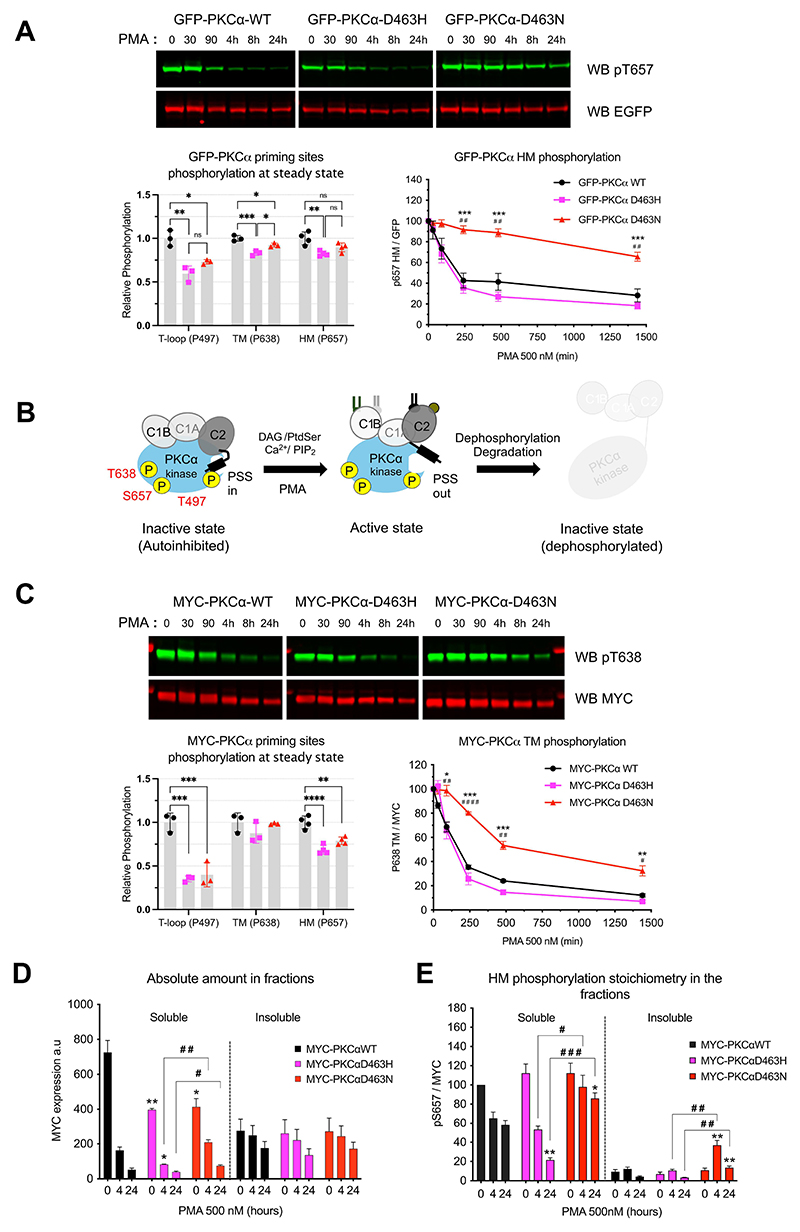
Regulation of PKCα D463H mutant is similar to that of WT-PKCα. **(A)** U87MG cells expressing transiently GFP-wild-type and mutant PKCα were treated with PMA for up to 24 hours. The blot at the top, quantified from *n* = 3 independent experiments in the bottom-left graph, shows the time course of the phosphorylation of the HM motif at Ser^657^ (pS657) and GFP-PKCα expression upon PMA was detected by Western blot with a phospho-specific antibody (green) and a GFP antibody (red). The data from the time course is presented as a percentage of decrease relative to the WT signal. An unpaired t-test was used to compare the HM phosphorylation of D463N vs D463H (*P* values*) or WT (*P* values^#^)(). Graph at bottom left shows analysis of the steady state phosphorylation of the three activation sites—T- loop at Thr^497^ (P497), TM at ^638^ Thr^638^ (P638), and HM at Ser^657^ (P657)— in WT and mutant PKCα prior to PMA stimulation. For the T-loop and TM analyses, n=3 independent experiments were performed, and *n* = 4 independent experiments were performed for the HM analysis. A one-way Anova was used to calculate the significance of the variation between WT and mutants. **(B)** Schematic representation of the life cycle of PKCα upon activation or PMA treatment. **(C)** As described for (A), with Myc-tagged PKC with and Myc antibodies. **(D and E)** Triton-X100 fractionation of U87MG cells expressing Myc-tagged WT and mutant PKCα following PMA treatment for 4 or 24 hours. The absolute amount (D) and the proportion of HM-phosphorylated (E) Myc-tagged protein in the soluble and insoluble fractions were analysed by Western blot. Data are from *n* = 3 independent experiments. Unpaired t-tests were used to compare WT to D463H or D463N (*P* values*) and D463H to D463N (*P* values^#^). ns, not significant; */^#^*P* < 0.05, **/^##^*P* < 0.01, ***/^###^*P* < 0.001, ****/^####^*P* < 0.0001.

To compare cellular behaviours of the PKCα WT and mutants, GFP-fusion proteins were expressed in U87MG cells, and characteristic activation-induced degradation monitored (Fig. 3, A and B). All three GFP-tagged proteins were phosphorylated at their priming sites (Fig. 3A), indicative of protein integrity, which is consistent with previously published findings for the D463N mutation (12). There was, however, a modest (about 25%) decrease in the phosphorylation of both mutants on the activation loop compared to WT (Fig. 3A) perhaps reflecting a more open conformation that we revisit below. The dephosphorylation and degradation of PKCα known to occur upon prolonged phorbol ester-dependent activation (Fig. 3B) was observed for WT and D463 mutants. Notably, the kinetics of the D463H mutant response mirrored that of PKCα WT whereas the D463N mutant was slower in the dephosphorylation of the HM motif (Fig. 3A), turn-motif, and the activation loop (fig. S4, A and B). The D463H mutant matched the WT in its degradation rate (fig. S4E). Similarly, the kinetics of induced dephosphorylation and degradation of the myc-fusion of the D463H mutant (myc-D463H) was alike those of the myc-WT, whereas those of the myc-D463N mutant were again slower (Fig. 3C and fig. S4, C and D).

The distinction between the mutants was also reflected in the detergent soluble versus insoluble distribution of proteins (Fig. 3D), where the D463N mutant was retained in the neutral detergent soluble fraction and was dephosphorylated more slowly in this context (Fig. 3E); in contrast, the D463H mutant behaviour closely aligned with the WT protein.

### Evidence for distinct protein interactions for PKCα D463H

**Fig. 4:**
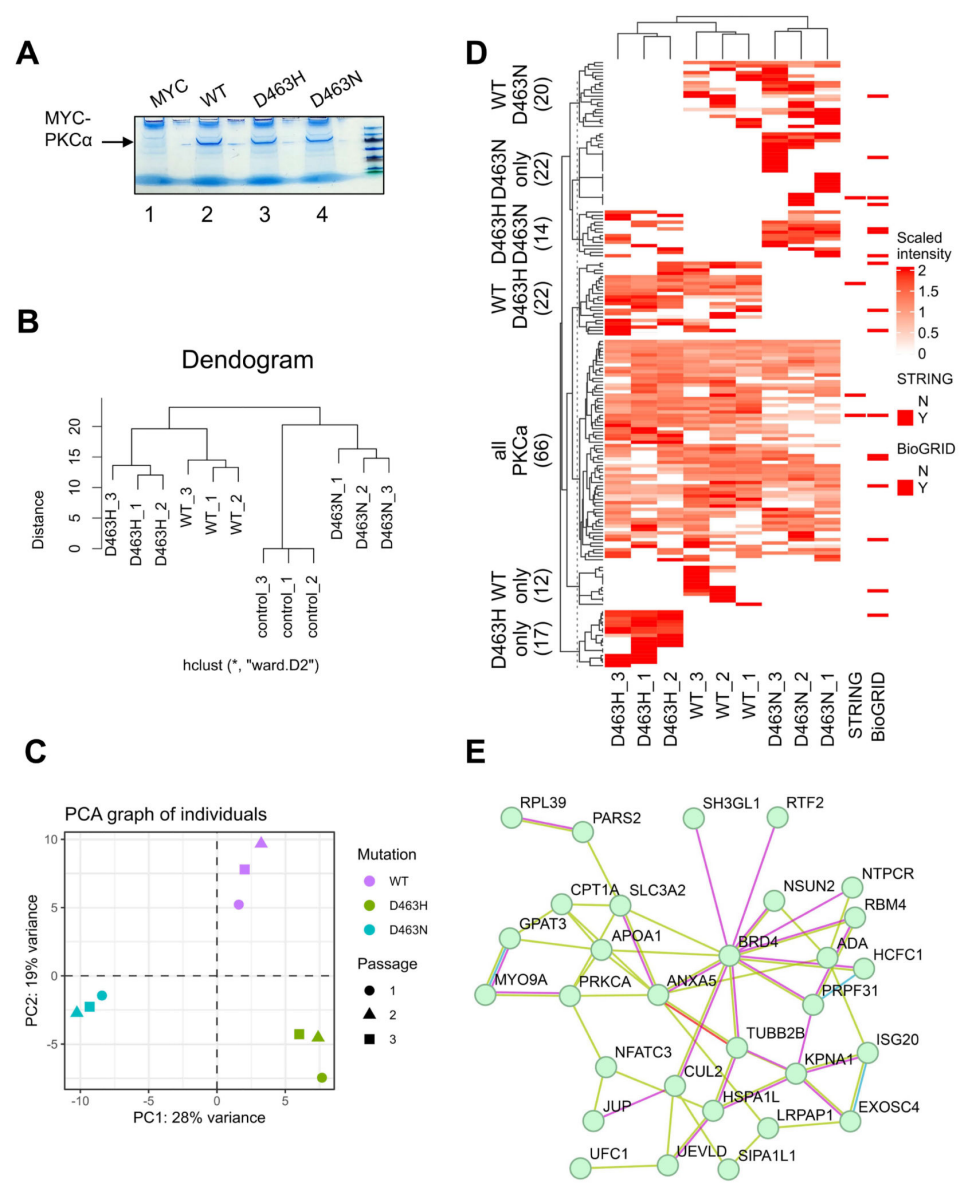
Proteomics of WT and mutant PKCα pull downs. **(A)** WT and mutant proteins as indicated were expressed as Myc-fusions in U87MG cells and captured on anti-MYC beads. Blot is representative of n=3 experiments. **(B)** Unsupervised hierarchical clustering analysis of mass spectrometry identified PKCα-co-precipitated proteins from stable U87MG cells expressing WT-PKCα, D463H mutant PKCα, or D463N mutant PKCα and controls (parental cells). “_1”, 2, and 3 indicate replicate samples. (**C**) Principal component analysis of the interactors found in PKCα WT and each mutant as described in (B). (**D**) Heatmap presenting the indicated patterns and overlaps of associated proteins identified as in (B). (**E**) STRING network of preferential D463H binders is displayed at 0.15 confidence level, demonstrating networking of key epigenetic regulators. Previously published interactions from experimentally determined (magenta) or curated datasets (cyan) are indicated, along with associations from text mining (green) and predicted interactions from gene fusions (red).

The distinctive in vivo behaviour of D463H with respect to WT and D463N led us to assess how this specific mutant might impose itself to trigger oncogenic signals in a non-catalytic fashion. To address this, we first sought to examine differences in the interactome between WT and D463 mutant PKCα in U87MG cells. U87MG cells were transfected with Myc-tagged WT-PKCα, PKCα-D463H, PKCα-D463N or empty-vector control as technical triplicates; all PKCα constructs expressed to comparable levels and were recovered equally in Myc immunoprecipitates (Fig. 4A), which were subsequently analysed by tandem mass spectrometry. Any proteins recovered in precipitates from empty-vector transfected cells were excluded as artefacts. A total of 200 proteins were identified as potential PKCα interactors, including known PKCα-interacting proteins caveolin (14), PKCγ (15), and annexin V (16). Principal component analysis and unsupervised clustering of interactomes indicates a clear distinction between the interactomes of WT, D463H and D463N mutants, with the D463H mutant interactome showing greater similarity to WT-PKCα (Fig. 4, B and C) than D463N. Approximately 90 proteins were identified as interactors with both WT and PKCα-D463H, with 15 showing statistically significant differences in their abundance between replicates (Fig.4D and data file S1). Furthermore, 11 high-confidence interactors were exclusively recovered with PKCα-D463H, including the oncogenic BET domain–containing epigenetic regulators BRD3:BRD4 (table S12). BRD4 has been implicated previously as a driver of glioma progression, potentially through promotion of MYC-driven anabolic pathways (see (17, 18)). To further explore PKCα functional roles for PKCα or the PKCα-D463H mutant in glioblastoma biology, STRING (19) analysis was conducted on proteins showing preferential binding to D463H over WT, which revealed networking between epigenetic regulators, including BRD4 and the MLL1 complex components HCFC1 (HCF-1) and PRPF31 (Fig. 4E). These pathways are associated with promotion of RNA polymerase II and MYC-driven transcription, through permissive histone modifications (20–25). These distinctive binding patterns suggest a gain-of-function for the D463H mutant and, notably, show clear differences between the D463H and D463N mutants.

We used TurboID-PKCα fusions (fig. S5A, B) in U87MG cells with or without PMA activation and biotin-streptavidin Western blotting to identify and analyze the protein interaction partners of the mutant. The fusion proteins themselves were biotinylated as expected (fig. S5C) alongside a substantial number of bands identified by the streptavidin probe (fig. S5D). This technique allowed us to detect a prominent biotinylated species of about 27 kDa in the D463H expressing cells that was absent from the WT cells under basal conditions but became biotinylated on stimulation with the PKCα activator PMA (fig. S5D). This is indicative of a specific upregulated interaction for the D463H mutant at steady state, which mimics the upregulated interaction observed on acute allosteric activation of the WT protein. Both the steady state interactome studies and the TurboID proximity analyses support the concept that D463H alters interaction of PKCα with binding partners to drive an augmented and functionally distinct oncogenic program. Enhanced association with epigenetic regulators also helps explain how a single point mutation might induce profound oncogenic reprogramming.

To explore whether structure predictions for PKCα can provide insights into the molecular details underlying this privileged behaviour, we used AlphaFold3 to assess the impact of a single-residue Asp^463^ substitution on its conformation. We predicted the structure of WT, D463H and D463N mutants, generating 100 models for each protein. For the WT, the top-scoring model displayed an auto-inhibited conformation, with the pseudosubstrate sequence reliably positioned docking into the active site in a substrate-binding mode (“PS-in”), and the C1a domain contacting αB, the αB-αC loop, and the C-tail (fig. S6A). The top-scoring model for D463N and D463H however displayed a confident prediction of an alternate “PS-out” conformation, with the C2 domain packing against the back of the C-lobe of the kinase domain (away from the active site) and the C-tail, and the C1 domains positioned under the kinase domain C-lobe and C2 domain. Mg^2+^ ions (one in D463N, both in D463H) were predicted outside of the active site and bound to the C2 domain, while the kinase domain was found in an intermediate open conformation with respect to N-lobe and C-lobe disposition, being more open than the nucleotide-bound WT prediction but not as open as the WT apo state. We then broadened the analysis to all predicted models, and this inspection indicated they all were either in a PS-in or PS-out state, so we first classified them based on their predicted conformation. For the WT protein, most models were predicted as PS-in, whereas for D463H and D463N, most were predicted as PS-out (fig S6B). We next compared model quality and characteristics of the predicted models by comparing the predicted Template Modelling (pTM) score and median predicted aligned error (PAE) score. The latter is used as a metric to compare the confidence in the relative positioning of domains within a predicted conformation. The mutant forms of PKCα showed better pTM scores and a better confidence in the PS-out conformation (fig. S6, C and D), with the best models observed for the D463H mutant. For the PS-in conformation, however, pTM scores were better for the WT. The D463N mutant showed a bimodal distribution, with about half of its PS-in models predicted with scores similar to those of WT or D463H (fig. S6E). Likewise, the confidence in PS-in conformers was worse for the mutants (fig. S6F). Overall, the WT protein is predicted as a PS-in conformer with high confidence, whereas the D463H mutant is predicted as a PS-out conformer with high confidence. These predictions suggest the D463H mutant presents as a PS-out, disinhibited state that is effector-competent, similar to an activated form of the WT protein, albeit incapable of ATP hydrolysis. The D463N mutant shows similar characteristics as the D463H mutant, although less pronounced, and its functional characterization supports a different nucleotide-binding profile that may not be compatible with effector trapping. The effect of D463 substitution is also supported by a predicted effect on stability of the WT kinase, which is more pronounced for D463H (Fig S6).

### PKCα D463H is a gain-of-function PKCα allele

**Fig. 5:**
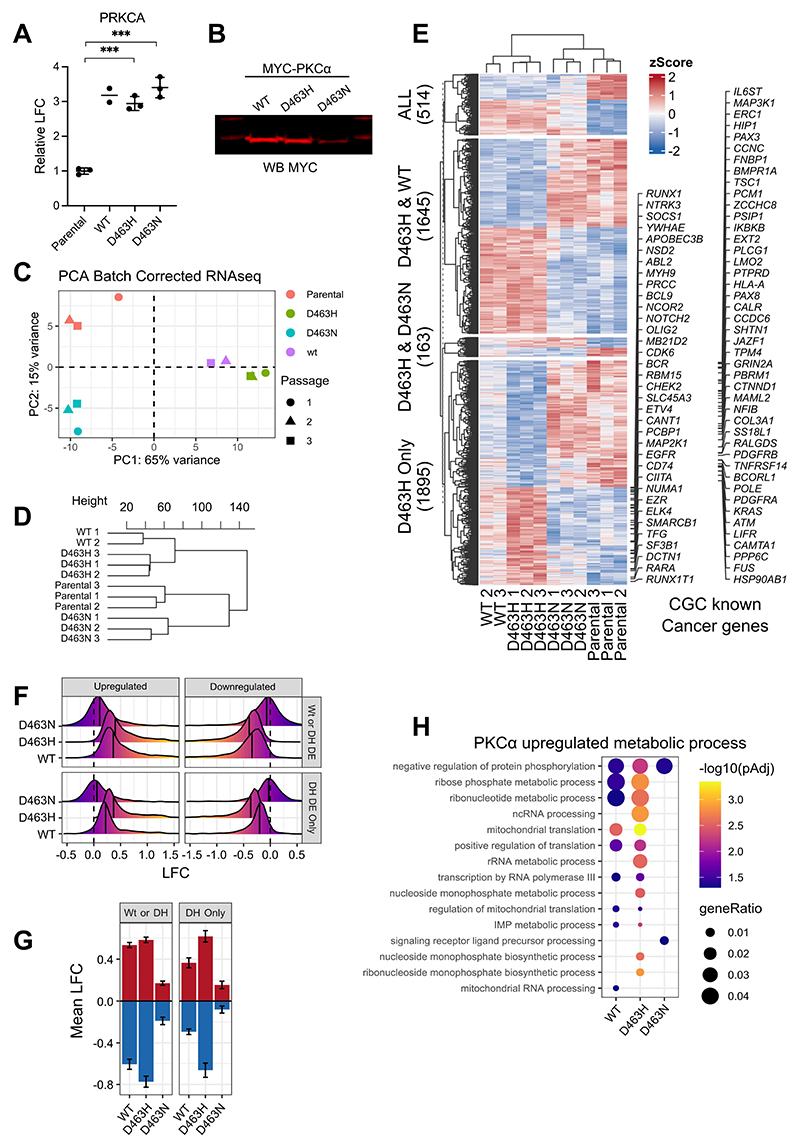
PKCα D463H is a dominant gain-of-function mutant which drives an augmented WT-PKCα transcriptomic signature. (**A**) mRNA expression of *PRKCA* in stable U87MG cells with Myc-tagged WT-PKCα, D463H or D463N mutants, as relative log fold change (LFC) to that of endogenous WT PKCα in parental cells, determined by RNA sequencing. Data are from *n* = 3 (parental and mutant cell lines) and n = 2 (WT). Statistical comparison to parental cells was performed using an unpaired t-test; ***P < 0.001. (**B**) Stable protein expression of Myc-tagged WT-PKCα and mutants D463H and D463N in stable U87MG by Western blotting using an antibody to Myc. Blot is representative of 3 independent experiments. **(C)** Principal component analysis of batch-corrected mRNA expression from individual experiments for the different stable cell lines expressing Myc-tagged WT-PKCα and mutants. **(D)** Unsupervised hierarchical clustering analysis of gene expression from all differentially expressed genes in stable U87MG cells expressing WT-PKCα, D463H-PKCα, or D463N-PKCα or parental cells. (**E**) Heatmap of genes differentially expressed upon overexpression of Myc-PKCα D463H (n=3 independent experiments) illustrating shared and exclusive patterns compared to WT, D463N and parental cell expression patterns. Tiles are coloured by per-gene z-scores across all samples. Rows are clustered by overlap in significant differential gene expression with WT and D463N samples when compared to parental cell lines. Cancer gene consensus (CGC)-derived “known cancer genes” are annotated for the D463H differentially expressed–only cluster. **(F and G)** Mean log fold change (F) and distribution of log fold change (G) for genes differentially expressed by WT or D463H mutant (Wt or DH DE; top in F, left in G) and by D463H mutant only (DH DE Only; bottom in F, right in G) relative to parental cells. **(H)** Overrepresentation analysis of the top eight statistically significant gene ontologies from the metabolic process (GO:0008152) gene ontology category child terms for the upregulated genes in stable U87MG cells expressing WT, D463H, or D463N PKCα parental cells.

Having established distinct biochemical characteristics and protein interactomes for the oncogenic PKCα D463H mutant, we next examined transcriptional changes in the U87MG models to explore mechanisms driving transformation. We compared U87MG glioma cells stably expressing WT-PKCα or the two D463 mutants. mRNA expression of PKCα was comparable between WT and the two mutants (Fig. 5A); however, the expression of D463N protein was reduced compared to WT or D463H (Fig. 5B). This may reflect differences in the protein stability or proteasomal targeting for the distinct D463 mutants. Nevertheless, all expressed proteins altered the pattern of mRNA expression and principal component analysis indicated tight clustering for biological replicates for each construct and the parental samples (Fig. 5C). Furthermore, all constructs resulted in significant displacement in principal component space away from the parental samples indicating that despite the difference in expression levels (in D463N versus the other two) there is a distinct impact of each PKCα allele on mRNA profiles.

Notably, the D463H mutant RNAseq data segregated closely with the WT and well away from the D463N mutant (Fig. 5C), consistent with similarities in biochemical behaviour and interactome changes. Unsupervised hierarchical clustering of gene expression profiles also indicates the close relationship of WT and the D463H mutant (Fig. 5D), which entirely mirrors that seen for the proteomic analysis (Fig. 4B). Whereas the majority of differentially expressed genes induced or suppressed by WT-PKCα follow the same directionality for PKCα D463H, the amplitude of change is generally greater for the D463H mutant; this can be seen in both principal component analysis, scatter analysis and through clustering of all differentially expressed genes when compared with parental (fig. S7, A and B). Exploring the amplitude differential between WT and the D463H samples, we separated gene clusters according to their significant differential expression patterns (relative to parental) between the WT, D463H and D463N experimental conditions (Fig. 5E). The largest individual cluster comprised 1895 genes which were significantly up- or down-regulated only by the D463H mutant; within this cluster, the vast majority (94.6%) of genes displaced from the parental in the same direction for both the D463H and WT-PKCα conditions, but the mean log fold change (LFC) for D463H was approximately doubled on average for the whole 1895-gene group (Fig. 5F). Examining the distribution of LFC clearly indicates the directional similarity but augmented response of the D463H compared with WT, in contrast to the distinct behaviour of the D463N mutant (Fig. 5G). Numerous known cancer-associated genes (of the cancer gene census, a component of the Catalogue of Somatic Mutations in Cancer (COSMIC)) were present within the D463H-only group that have augmented gene expression (Fig. 5G). The second largest cluster comprised genes which were differentially expressed upon expression of either WT or D463H, and these largely show parallel behaviour but strong differential expression compared with D463N and parental cells (Fig. 5E). These dominant groups underly the directionality and amplitude of movement observed in the principal component analysis and also identify a specific gene-expression output that was noticeably augmented by the D463H mutant. To assess functional impact, we conducted gene set over-representation analysis and focused on gene sets associated with translation and metabolism. The results (Fig. 5H) suggest that D463H may act as a gain-of-function mutant, augmenting expression of multiple anabolic gene sets compared to WT and eliciting an entirely distinct pattern compared to the loss-of-function mutant D463N.

Only a minor group of 163 genes showed some degree of similarity between the two kinase-inactive D463N and D463H mutants compared with WT (Fig. 5E). We would interpret these alterations as those where PKCα kinase catalytic activity plays an active effector role. Overall, however, these data suggests that PKCα kinase activity is of minor importance to ‘positive’ effector outputs in the context of this glioblastoma derived cell line, consistent with the in vivo observations.

**Fig. 6:**
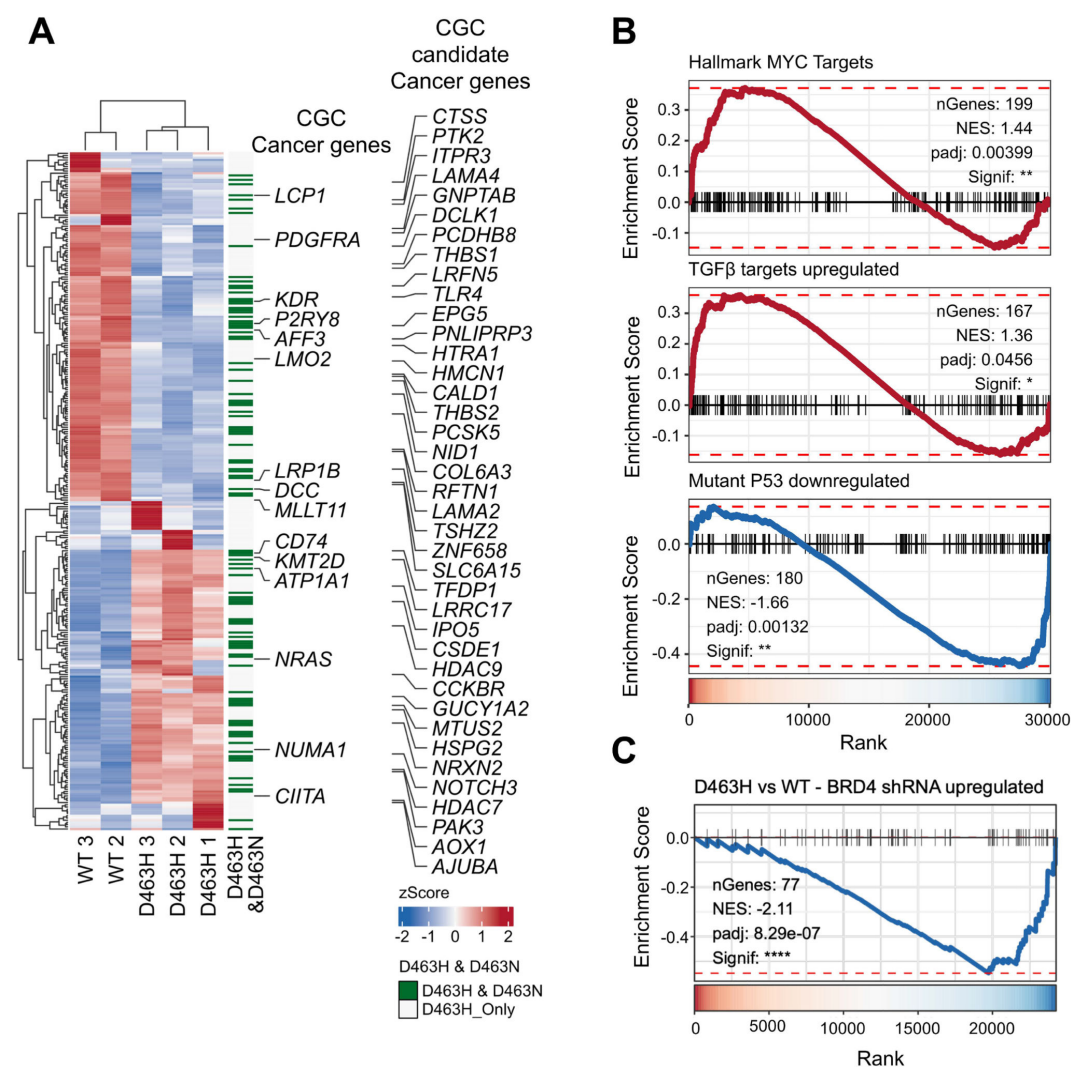
Differential gene-expression induced by WT-PKCα and PKCα D463H. **(A)** Heatmap of genes differentially expressed between U87MG cells stably expressing PKCα D463H (n=3 experiments) or WT PKCα (n=2 experiments). Heatmap annotation (green lines) identifies overlap in differentially expressed genes between PKCα D463H and D463N-expressing cells relative to WT expressing cells. Known and candidate cancer genes determined by the Cancer Gene Consensus (CGC) database of cancer genes are annotated with corresponding differential gene expression significance. *P<0.05, **P<0.01, ***P<0.001, ****P<0.0001 **(B)** Enrichment plots from GSEA analysis for hallmark MYC pathway target genes, upregulated TGF-β target genes, and downregulated p53 target genes obtained from the molecular signatures database. Ranks were calculated based on the Wald statistic from differential expression results between PKCα D463H and WT-expressing cells. *P<0.05, **P<0.01. **(C)** Enrichment plot from GSEA for a gene set comprising upregulated genes in U251 glioblastoma cells targeted with a BRD4 shRNA previously published. Ranks were calculated based on the Wald statistic from differential expression results between PKCα D463H and WT-expressing cells. ****P< 0.0001 by GSEA test.

Among the D463H differentially expressed genes (Fig. 6A and fig. S7) there were many known and candidate “cancer genes” (again, of the Cancer Gene Census from COSMIC), including enhanced expression of *NRAS, KMT2D*, and *CD74*. The majority of these genes were not differentially expressed between WT and D463N cells. Gene set enrichment analysis (GSEA) also revealed potential phenotypic differences promoted by D463H compared with WT; TGFβ- and Myc-target signatures are increased, whereas mutant p53–downregulated genes were suppressed (Fig. 6B). These are pathways that are broadly implicated in most human tumours, including glioma (26–29). D463H also enhanced a signature associated with cyclin D-mediated cell cycle progression (30), which has previously been reported as a downstream growth effector axis for PKCα in glioma (fig. S8A) (31–33). Bioinformatic analysis did not, however, implicate PKCα D463H in the regulation of common oncogenic signalling pathways, suggesting a non-canonical signalling mechanism underlying deregulation of Myc, p53, and cell-cycle progression (fig. S8A). Expression of D463H also enriched the signatures for anabolic processes, including protein translation, amino-acid and nucleic acid metabolism, and ribosome biogenesis—all in line with enhanced Myc-driven growth (fig. S8, B and C).

**Fig. 7:**
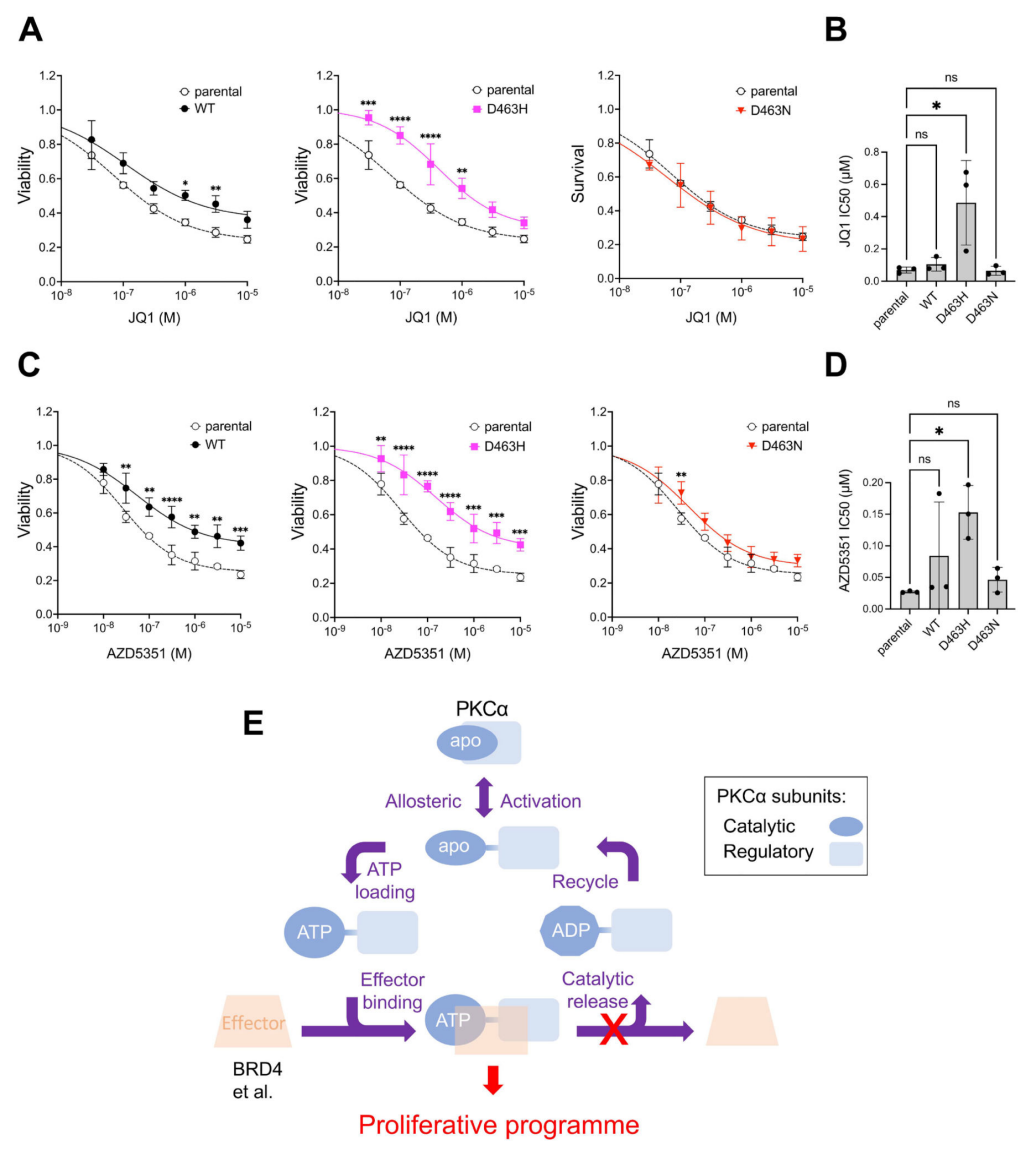
Resistance to BET inhibitors of PKCα D463H mutant expressing cells. (**A to D**) Viability of U87MG stably expressing WT-PKCα, D463H and D463N and treated with increasing concentrations of JQ1 (A and B) or AZD5351 (C and D). Data are derived from n=3 independent experiments. Each cell line is separately compared with the same parental U87MG cell line. Dose response curves (A and C) represent the mean viability from three independent biological replicates +/- S.D., with significance assessed using two-way ANOVA with Tukey’s multiple comparisons. IC_50_ values (B and D) were calculated separately for each biological replicate, and data are presented as the mean +/- SD, with significance assessed by one-way ANOVA compared to the parental control. *P* < 0.05 (*), *P* < 0.01 (**), *P* < 0.001), *P* < 0.0001 (****). **(E)** Schematic of PKCα and D463H mutant kinase-independent signalling. In the basal state (for simplicity an apo form) PKCα cannot interact with downstream effector(s). On allosteric activation triggering an open conformation, the kinase domain can load ATP, and the ATP-bound state is competent to bind downstream effector(s). In some specific cellular contexts, the formation of a PKCα/effector complex drives a proliferative signal which is switched off through kinase activity-dependent effector release. In the case of the loss-of-activity, gain-of-function D463H mutant that retains an ATP-bound active conformation, the lack of catalytic activity prevents the normal physiological release of the effector (red cross), sustaining complex formation and a persistent proliferative output.

The enhanced interaction of PKCα-D463H with epigenetic regulators provides a potential mechanism driving observed changes to the transcriptome. Intriguingly, the D463H binder BRD4 has been implicated as an oncogenic regulator of many cancers, including glioblastoma, and is widely linked with Myc-driven tumorigenesis (18). Usefully, Du *et al*. conducted a study examining BRD4-dependent gene-expression in U251 glioblastoma cells (34). Using gene sets derived from this data and GSEA analysis, we showed that genes upregulated by BRD4 loss are significantly negatively enriched following expression of PKCα-D463H (Fig. 6C). This supports the concept that PKCα-D463H may regulate transcription through targetable epigenetic mechanisms, providing a potential clinical strategy for *PRKCA* mutant choroid glioma, particularly as BET domain inhibitors have been proposed for use in other glioma settings (35). We therefore sought to examine the impact of PKC mutant over-expression on sensitivity of U87MG cells to the BET inhibitors JQ1 and AZD5351. Over-expression of D463H, but not D463N, resulted in significantly decreased sensitivity to growth inhibition in response to either JQ1 or AZD5351 (Fig. 7, A to D). D463H increased the target efficacy (inferred from half-maximal inhibition (IC_50_) value) of both JQ1 and AZD5351 by approximately 5-fold relative to parental and WT- or D463N-expressing cells (Fig. 7, B and D). Notably, although WT-expressing cells consistently showed lower growth inhibition with JQ1 and AZD5351, IC_50_ values did not significantly change relative to parental cells (Fig. 7, B and D).

## Discussion

The early onset chondrosarcomas associated with the heterozygous knock-in of the penetrant chordoid glioma-associated *PRKCA* D463H mutation in mice and the lack of any phenotype associated with the related D463N mutant knock-in suggests that this mutation confers an in vivo gain-of-function. The allied biochemical, interactome and transcriptomic analyses associated with this mutant, suggests that PKCα D463H promotes a non-canonical cell-cycle progression and pro-growth phenotype, resembling an augmented PKCα driven response, driven through epigenetic pathways. Mechanistically, this gain-of-function and loss of catalytic activity suggests a working model of effector complex-driven output under negative feedback control of catalytic activity (Fig.7E).

Neonatal morbidity of the *PRKCA* D463H mutant mice precluded assessment of any slow growing chordoid glioma, nevertheless, the parsimonious conclusion would be that the driver behaviour of this mutation is conserved in the context of β tanycytes with implications for non-surgical intervention. The model suggests that the PKCα D463H mutant preferentially resides in a conformational state, capable of promoting tumour development. This and previous studies indicate that high levels of PKCα, or PKCα retained in an active configuration are oncogenic in glioma, but that kinase activity is not required (12, 36). This provides some explanation for why ATP competitive inhibitors of PKCα have shown no benefit in the clinic to date (1); on the contrary inhibitors may provoke PKCα output by locking a signalling competent conformer, as promoted by the D463H mutation.

Evidence on the underlying properties associated with PKCα mutant function have come through multiple avenues contrasting the behaviour of WT and D463H (phenotype inducer) with that of D463N (phenotype neutral) mutants. Comparison of nucleotide binding affinities suggest that D463H preferentially binds ATP over ADP, and with ATP affinity much higher than the D463N mutant. We propose a mechanism where the combination of both ATP binding preference and lack of ATPase activity would benefit an ATP occupied active conformation for D463H-PKCα. Structural modelling suggests both D463H and D463N mutants exist in a PS-out, activated conformation. This effector-competent conformation of the D463H mutant is further supported by the Turbo-ID profile. This marks D463H out as a privileged gain-of-function mutation analogous to gain-of-function small GTPase mutations.

We infer that intervention in chordoid glioma would be effective through the elimination of mutant PKCα protein. The PKCα-directed antisense agent aprinocarsen has not shown efficacy in other tumour types (see (37)), however the application of targeted protein degraders offers a powerful alternative (for review (38)). Targeting oncogenic effectors downstream of PKCα may also provide therapeutic options. The enriched association of epigenetic regulators with PKCα D463H, including BRD4 and MLL1 complex components, warrants closer attention, given that licenced drugs are available for both. Indeed, the enhanced signatures for c-Myc and BRD4, and the functional protection against two distinct BET inhibitors, driven by D463H over-expression in glioma cells, provides strong evidence that this mutant preferentially interacts with the BRD4 axis. BRD4 and related BET domain family members, alongside HCFC1, are potent promoters of RNA polymerase II and Myc driven transcription (see for example (23, 24)), offering a likely underlying mechanism. Targeting BET-domain family members has been widely proposed as an approach for suppressing Myc driven tumour growth, including in neuroblastoma and glioma settings (17, 18, 39–42). Revumenib which targets MLL1 (also known as KMT2A) has recently been approved for a series of pathway modified tumours (see (43)). Studies to examine the therapeutic potential of epigenetic targeting drugs in PKCα-D463H mutant choroid glioma are warranted.

Chondrosarcomas are bone malignancies that derive from chondrocyte and chondroblast progenitors (see (44)), characterised by the production of cartilaginous extracellular matrix, with a low percentage of dividing cells, as reported here for D463H mice hindlimbs. The Archs4 database (https://maayanlab.cloud/archs4/) indicates that PKCα is highly expressed in chondrocytes and is linked to an abnormal chondrocyte physiology in mice (MP0009780). There is also involvement of PKCα in the regulation of extracellular matrix organization (GO:1903055) chondrocyte proliferation (GO:0035988) and cell adhesion (GO:0033627) (https://maayanlab.cloud/archs4/gene/PRKCA). In concordance, our GSEA analysis of the canonical pathways also showed a decrease in the extracellular matrix organisation, osteogenic collagen synthesis and integrin signalling associated with PKCα D463H expression. This suggests some canonical effects of this specific PKCα D463H mutation may be shared despite the distinctive cellular contexts.

Chondrocytes translate mechanical forces to regulate cartilage homeostasis and function (45) and the pathogenesis of bone and cartilage are linked to alterations in these mechanotransduction pathways (reviewed (46)). PKCα was shown to be important in mechanotransduction signaling downstream of integrins in chondrocytes (45) through a role for the PKCα/ERK-1 axis (47), consistent with findings in chordoid glioma (5) and here. The results suggest that the formation of chondrosarcomas in the hindlimbs of D463H mutant mice might be due to PKCα D463H driven dysregulation of the mechanotransduction signals involved in cartilage homeostasis.

Various kinase inactive PKCα mutants have been employed in different studies to examine their dominant negative impact on physiology; our results suggest that the preconceived expectation of their impact needs reassessing. For example, it has been shown that expression of the inactive PKCαK376R mutant in haematopoietic progenitor cells which are then differentiated down a B-Cell lineage, triggers the formation of a B-CLL phenotype (48). The original interpretation promoted a suppressive function in B-cells, re-evaluation considering the results here might lead to a rather different conclusion. Generally, the penetrance of catalytic activity independent properties of PKCα and indeed other members of this family and the wider kinome would merit broad consideration. In the context of pseudokinases we readily accept the importance of allostery and the associated influence of ATP binding on kinase domain conformation (see (49)). The implication of the current findings is that such properties can be ascribed to active kinases.

The evidence presented provides a paradigm shift in our appreciation of PKCα action and questions our understanding of the wider kinome. Unravelling the conformation-dependent but kinase-independent proximal targets and associated downstream programs elicited by this PKCα mutant, will provide insights that will no doubt guide the development of innovative interventions.

## Materials and Methods

### Antibodies and Reagents

Phospho-PKC Substrate Motif [(R/K)XpSX(R/K)] MultiMab™ Rabbit monoclonal antibody mix (#6967), phospho-(Ser) PKC substrate antibody (#2261), phospho-PKC (pan) (gamma Thr^514^) antibody (#9379), phospho-PKCα/β II (Thr^638/641^) antibody (#9375), phospho-PKC (pan) (βII Ser^660^) antibody (#9371) and PKCα antibody (#2056) were from Cell Signaling Technology. IRDye800CW Streptavidin (926-32230), anti-rabbit IRDye800CW (925-32211), anti-mouse IRDye680RD (926-68070), and Odyssey Blocking Buffer (TBS) were from Li-COR Biosciences. Antibodies to HA tag [HA.C5] (AB18181) and Myc tag [9E10] (ab32) were from Abcam. SYPRO orange protein gel stain (S5692) was from Sigma-Aldrich. Mouse monoclonal antibody to GFP [9F9.F9] (Ab1218) was from Abcam. InSolution PMA (phorbol 12-myristate 13-acetate) was from Merck. FuGENE^®^HD (E2312) was from Promega. cOmplete™, EDTA-free Protease Inhibitor Cocktail (12326400) was from Roche. Streptavidin-HRP conjugate (GERPN1231) was from Sigma-Aldrich. Lipofectamine RNAiMAX Transfection Reagent (13778150) was from Life Technologies LTD. HA-probe (F-7), monoclonal antibody (sc-7392), was from Santa Cruz Biotechnology. Complete mini EDTA free (4693159001) was from Sigma-Aldrich. The RNeasy Mini Kit (74104) And the QuantiTect SYBR Green RT-PCR Kit (204243) were from Qiagen LTD. ADP-Glo™ kinase assay reagents (V6930) were from Promega. The In-Fusion cloning kit was from Takara (638948). Phosphatase inhibitors set II (524625) and set IV (524628) were from Millipore.

### CRISPR/Cas9 genome editing for *Prkca* D463H knockin mutation in mice

All animal studies were compliant with UK Home Office regulations and carried out under license. *Prkca* protein kinase C, alpha [*Mus musculus* (house mouse)] - Gene ID: 18750 was targeted by CRISPR. The CRISPR-Cas9 reagents were delivered directly into the mouse zygote to derive a mutant mouse carrying targeted genetic modifications. The single guide sgRNAs, which provide target specificity for D463 > H mutation in exon 13 on chromosome 11, were Guide RNA 1 and Guide RNA 2. The protocol uses ZEN, 1.2 µM/6 µM/300 ng/μL, N, 2c, Repair – x1 ssODN, 75, Guide1 and ZEN, 1.2 µM/6 µM/300 ng/μL, N, 1c, Repair – x1 ssODN, 75, Guide 2 respectively was used for 107 embryo injections in total. The Cas9 nuclease specificity that creates the DNA double-strand break is dictated by the PAM motif a trinucleotide sequence of NGG, directly adjacent to and continuous from the 3’end of the protospacer sequence of the noncomplementary strand. The donor oligonucleotide or plasmid carrying the intended mutation flanked by sequences homologous to the target site were injected with the guide RNAs (table S10).

### Mouse cohorts

Mouse strains with D463H mutation were: IRCP 1, 3 and 4, mosaic mice C57BL/6Jax (table S1); PPBR 5-10, Het mice C57BL/6Jax x C57BL/6Jax cross (table S2); PPBY 1, 4 and 5: Het mice C57BL/6Jax x CD-1 cross (table S3); PPBY 6 and 7, Het C57BL/6J x CD-1 cross in trio with CD-1 (table S4). The second round of CRISPR for the D463H mutation were: IRCP 8, mosaic mice with C57BL/6Jax background; IRCP 20 mosaic mice with F1(B6 x CBA) background; PPCE 1 and 2: Het mice C57BL/6Jax x CD-1 cross (table S5); PPCE 7 and 8: Het mice F1(B6 x CBA) x CD-1 cross (table S5). Mouse strains with D463N mutations were: IRCY 3 and 6, mosaic mice with C57BL/6Jax background (table S6); IRCY 11, 12 and 13, Het mice C57BL/6Jax x C57BL/6Jax cross (table S7); PPBZ 2 and 3, PPCA 1, PPCA 11 and PPCB 1, Het mice C57BL/6Jax x C57BL/6Jax cross (table S8); PPCA 4, Het mice C57BL/6Jax x CD-1 cross; PPBZ 7, Het mice C57BL/6Jax x F1(B6 x CBA) cross (table S8). PPBZ 8 and PPCB 12, homozygous mice C57BL/6Jax x C57BL/6Jax cross (table S9). No phenotypic variation was observed between the sexes.

### Histopathological Examination

Formalin-fixed, paraffin-embedded (FFPE) sections, each 4-µm thick, were stained with haematoxylin & eosin (H&E) and examined by two board-certified Veterinary Pathologists (A.S.-B. & S.L.P.). Histopathological assessment was performed blind to experimental grouping using a light microscope (Olympus BX43). Tissue sections were examined individually and in case of discordance in diagnosis a consensus was reached using a double-headed microscope. Sections were histopathologically assessed using the INHAND guide for non-proliferative and proliferative lesions of the rat and mouse skeletal tissues (50).

### Recombinant protein production in insect cells

For the generation of the baculovirus, Sf21 insect cells were co-transfected with the pTRI-EX vector containing PKCα and FlashBAC™ backbone baculovirus vector. The viruses were amplified by a series of 0.22 µm filtered viral supernatant infection of Sf21 cells. The extraction of the recombinant proteins was done by resuspending the spun cells in wash buffer: 50 mM Tris-HCL pH 7.5, 150 mM NaCl, 1 mM EDTA and 1 mM DTT containing 1% Triton X-100 with addition of protease inhibitors for lysis. The centrifugated lysate supernatant was put on Glutathione-Sepharose beads 4B and left to bind for 2 hours on roller at 4ºC and the beads washed 3 times with the wash buffer. For elution of the GST-proteins, the beads were resuspended in wash buffer containing 20 mM Glutathione reduced (pH 7.5) and left over/night at 4ºC in slow motion roller/rotisserie. For 3C or TEV cleavage, the beads were re-suspended in wash buffer with 3C-GST or TEV-GST proteases, and the cleaved recombinant proteins were recovered by centrifugation. For larger scale protein extraction, the same protocol was used, and the proteins were spun at 4,000 rpm at 4ºC down to 500 µl using a Vivaspin 6 concentrator (30,000 cut off for full-length PKC and 10,000 cut off for kinase domain). The proteins were purified on Superdex S200 AKTA. The correct expression of GST-PKCα full length and kinase domain was confirmed by SDS-PAGE Coomassie gels and their phosphorylation by Western blot using anti-phosphospecific antibodies.

### Pull down GFP-DARPin and kinase assay

All cell lines were authenticated by short tandem repeat (STR) profiling and Mycoplasma screened by a PCR-based approach by Cell Services at The Francis Crick Institute. U87MG transiently transfected with GFP-PKCα and mutants were solubilised in lysis buffer (20 mM Tris, pH 8; 130 mM NaCl supplemented with 1% Triton X-100). GFP-PKCα constructs were pulled down using DARPin (Designed Ankyrin Repeat Proteins) agarose beads directed against GFP and subjected to a kinase assay using 20 mM TRIS pH 8, 2 mM DTT, 100 nM Calyculin A, 10 mM MgCl2, 1 mM ATP supplemented with PKC activators: 400 µg/ml PtdSer, 1 µM PMA, 300 µM CaCl2. GFP-PKCα and associated substrates phosphorylation were detected by western blot using anti PKCα phospho-substrates and anti GFP antibodies.

### Fractionation assays

After treatment with vehicle (0 hours) or 500nM PMA for 4 or 24 hours, U87MG cells were solubilised in 20 mM Tris, pH 8, 130 mM NaCl, 1% Triton X-100 supplemented with protease inhibitors for 15 min at 4°C. the cells were scraped and put in an Eppendorf and centrifuged at 16,000g for 10 min at 4°C. LDS/DTT (4X) was added to the supernatant (soluble fraction) and the pellet (Insoluble fraction) was diluted in (2X) LDS/DTT for loading on an SDS-PAGE gel. The proteins were transferred onto a PVDF membrane and the amount of PKCα in each condition was detected by western blot using an anti-HA antibody. The phosphorylation of PKCα hydrophobic motif over total protein was detected using an antibody to phosphorylated Ser^657^ (pan-PKCβ^660^; Cell Signalling Technology) concomitantly with an antibody to HA tag (Li-COR).

### RNA sequencing and analysis

Stable U87MG cell lines expressing Myc-PKCα WT, D463H and D463N and parental control cells were grown and plated on a 6-well plate to isolate mRNA. The RNA extraction was done as per manufacturer protocol (RNeasy kit from Qiagen) on about 1 to 1.5 M cells. The mRNA from 3 independent experiments were sequenced and analysed for each construct by the high throughput sequencing facility technology platform at the Francis Crick Institute. Differential expression analysis was conducted using the R package DESeq2 (51) and batch correction of counts using the R package limma (52). Bioinformatics was performed using bespoke R scripts. Known and candidate cancer genes were obtained from the Cancer Gene Consensus (CGC, COSMIC) (53). Overrepresentation analysis was performed using the R package clusterProfiler (54) using ontologies obtained from the Gene Ontology (GO) resource (55). Geneset enrichment analysis (GSEA) was performed using the R package fgsea (56) using genesets curated from the molecular signatures database (MSigDBv7.5.1). The BRD4 shRNA–upregulated geneset was curated by extracting the top 100 upregulated genes upon BRD4 shRNA treatment in U251 cells in a previously published data set (GSE97791, GEO). Custom R scripts used to analyse and plot data are publicly available at https://github.com/jackchenry/PKCa_Calleja-et-al.

### TurboID experiments

Stable U87MG cells expressing constitutively 3xHA-TurboID-PKCα fusion constructs WT and mutants were plated on a 6-well plate at 500,000 cells/well. Biotin (500 µM) was added the day after for 10 to 30 min. The cells were then washed extensively to remove the free biotin, lysed with RIPA buffer (50 mM Tris-HCl, pH 7.4, 150 mM NaCl, 0.5% deoxycholic acid, 1% NP-40, 1 mM EDTA) supplemented with 0.1% SDS and protease inhibitors cocktail (cOmplete™ mini from Roche) and pulled down with neutravidin agarose beads (Thermo Fisher Scientific). After 3x washes using the lysis buffer at 4ºC the beads were recovered in sample buffer and the proteins separated by SDS-PAGE. After transfer onto PVDF membrane the biotinylated proteins were detected by western blot using streptavidin labelled with an IRDye 800 (LI-COR/Odyssey detection system). For simple detection of protein expression upon various treatments the stable cell lines were directly lysed by addition of Nu-PAGE LDS sample buffer (Thermo Fisher Scientific) supplemented with DTT (1/10e) separated by SDS-PAGE and detected by western blot with streptavidin-IRDye800. In parallel the expression of the PKC constructs was verified by western blot with an anti-HA antibody and a secondary IRdye680 (red pseudocolour).

### Thermal shift assay

The thermal shift assay is an indirect measurement of protein stability. Upon increase in temperature proteins unfold allowing the binding of the fluorescent dye SYPRO orange. The increase in fluorescence versus temperature is recorded in real time (melting curves). The stability of PKCα and mutants was measured in the apo form or when stabilized with addition of increasing amount of ATP or ADP (15 dilutions ranging from 0.1 µM to 5 mM). On a 384-well plate each condition was acquired in triplicate, 0.4 to 0.7 µg of recombinant protein PKCα WT or mutants was added without or with a serial dilution of ATP or ADP ranging from 0.01 mM to 5 mM. The proteins and nucleotides were diluted in the thermal shift buffer containing 50 mM HEPES, 150 mM NaCl, 1 mM DTT supplemented 10mM MgCl_2_. SYPRO orange (1:1000 in thermal shift buffer 1X) was then added to the mix. The plate was placed in a QuantStudio7 flex RT-PCR with Melt curve standard program gradually increasing the temperature from 15°C to 75°C with an incrementation of 0.5ºC/s. The melting curves (ascending part of the curve) were fitted with a non-linear sigmoidal or a biphasic fit using GraphPad/Prism software at each concentration of ATP or ADP to determine the melting temperatures (T_m_). The stabilization effect (ΔT_m_) of the ligand for each protein was subsequently obtained by subtracting T_m_ [ligand] – T_m_ [apo] at each concentration of ATP or ADP. The EC50 were obtained by plotting the ΔT_m_ versus [ATP] or [ADP] and fitting the curve with GraphPad/Prism.

### PKC recombinant protein labelling via amine coupling with NHS RED dye for MST analysis

The labelling of the PKCα full length recombinant proteins was done using Monolith protein labelling kit RED-NHS 2^nd^ generation (Nanotemper) according to manufacturer’s protocol. Briefly, the recombinant proteins were first buffer exchanged into 50 mM Hepes, 150 mM NaCl buffer prior to labelling to exchange from the original buffer containing Tris. Proteins and dye were incubated for 30 min at room temperature. The excess dye was removed using a spin column. The proteins were eluted in 450 µl of MST buffer 50mM Hepes, 150 mM NaCl, 10 mM MgCl_2_ and fresh 1 µM DTT. The aim was to obtain a ratio of dye to protein in the order of 0.3 to 1 for the MST experiments.

### Microscale thermophoresis (MST)

PKCα full length recombinant proteins labelled with NHS RED and diluted in the MST reaction buffer were mixed with ATP at various concentrations. For each assay, 12 x 20 µl samples containing the recombinant proteins at a final concentration of 50nM or 100nM as used. 20 µl of ATP was added to the reaction in a 2-fold serial dilution as indicated in the graphs. Samples were transferred to premium Monolith NT.115 capillaries. Experiments were run with a LED power of 20 % and MST power of 20 %, at 25°C, with a 5s-20s-5 run. Run on Monolith NT.115 (Nanotemper technologies) machine, using NT Control v.2.2.1 (Nanotemper Technologies) software and analysis performed in MO Affinity Analysis v.2.3 (Nanotemper Technologies) software. The Kds were determined by fitting the data to a non-linear regression using a quadratic binding model.

### Cloning of the PKCα constructs

pEGFP-C1-PKCα (GFP-tagged human PKCα) is an in-house construct (The Parker laboratory at the Francis Crick Institute). Constructs were created by InFusion® cloning in vectors cut with EcoRI. pCDNA3-Myc-PKCα was performed by cloning PCR amplified PKCα with an N-terminal Myc tag using the oligos Myc-PKCα_sense and _anti. pBABE puro-Myc-PKCα was done by cloning of PCR amplified Myc-PKCα using the oligos Myc-PKCα_for and _rev. pBABE puro-3xHA-TurboID-PKCα was done by cloning PCR amplified 3xHA-TurboID from the vector Addgene 3xHA-TurboID (#107171) upstream of the 6xGly-PKCα PCR product using the oligos 3xHA-TurboID_for and _rev and 6xGly-PKCα _for and _rev. pTriEX6-GST-PKCα insect cells expression construct was done by cloning the PCR product of full length PKCα into the vector cut with EcoRI-BamHI, using the primers GST-3C PKCα_for and _rev. pTriEX6-GST-PKCα kinase domain was done by introducing a TEV cleavage site (E-N-L-Y-F-Q-|-G/S, cleavage between Q and G/S) in the hinge region just after the residue 321 of full length PKCα in the pTriEX6-GST-PKCα construct. PKCα regulatory domain, a fragment containing the TEV site, and PKCα kinase domain with the C-terminal were PCR amplified separately. The three PCR products were introduced into pTriEX6-GST-3C cut with EcoRI-BamHI. The oligos used for the 3 PCR amplifications are listed in table S11.

### Stable Cell lines

To generate stable polyclonal cell lines expressing Myc-PKCα for the RNAseq experiments, U87MG cells were plated in a 6-well plate at 300,000 cells per well and transfected 24 hours later with pBABE puro-Myc-PKCα WT or mutants. The cells were then selected with puromycin until the control non-transfected cells were all dead (approx. one week). To prepare stable polyclonal cell lines expressing 3xHA-TurboID-PKCα WT and mutants for the identification of protein partner patterns, cells were transiently transfected with pBABE puro-3xHA-TurboID-PKCα and mutants then selected with puromycin until the control non-transfected cells were all dead, approximately one week later.

### PMA-induced PKCα degradation experiments

U87MG cells expressing transiently GFP-PKCα or Myc-PKCα WT and mutants were treated with 500 nM PMA for various durations up to 24 hours. The time course decrease of PKCα priming site phosphorylations were detected by western blot using anti phospho-specific HM, turn motif and activation site antibodies, and the expression of each protein determined with anti GFP or anti Myc antibody.

### Kinase activity of recombinant PKCα full length or kinase domain

After determination of the optimal PKCα or PKCα kinase domain concentration (linear range of protein v activity), the Michaelis-Menten constant (K_m_) for ATP was determined by dose response of ATP. 50 ng of recombinant enzyme was mixed with 25µM ATP (approx. K_m_) and with activators in kinase buffer in the presence or absence of substrate either 0.2 mg/mL peptide substrate (PPSS) or 0.5 mg/mL of Protamine sulphate (no activators required). The kinase buffer (1X) was prepared as follow: 40 mM Tris/HCL pH 7.5, 20 mM MgCl_2_, 0.1 mg/mL BSA and 50 µM DTT. The activators 400 µg/mL of phosphatidylserine (PtdSer) in 1% TX-100, 1 µM PMA and 300 µM CaCl_2_ were added only with the PPSS peptide (Ac-RFARKG**S**LRQKNVH-CONH2). The reactions were performed as specified by the manufacturer’s protocol (ADP-Glo™ - Promega).

### Preparation of the samples for immunoprecipitation and mass spectrometry

U87MG cells were plated at 500, 000 cells per well on a 6-well plate. The following day the cells were transfected with 2 µg/well of Myc-PKCα WT or D463H or D463N mutants. On day 4, the cells were lysed in 250 µl/well of lysis buffer containing 20 mM Tris pH 8.0, 130 mM NaCl, 1% Triton X-100, 1 mM DTT, 10 mM NaF with protease and phosphatase inhibitors (ROCHE). 3 wells were used per condition. The immunoprecipitation was performed using Myc-trap®Agarose (ChromoTek). After 3 washes with lysis buffer without inhibitors, a bead-associated kinase assay was done on the IPs with 1mM Mg-ATP, in a total volume of 50 µl of 20 mM Tris/HCL pH 8, 10 mM MgCl_2_, 100nM CalyculinA and 2mM DTT. The supernatant was recovered 50 µl and 20 µl of LDS 4X/DTT was added. The beads were recovered in 50 µl of LDS 2X. The proteins were separated after a short run by SDS-PAGE on a 10 % Bis-Tris gel. The gel was stained with Coomassie blue, and the bands were cut and processed for mass spectrometry. A Western blot (Li-COR Odyssey^®^) with total Myc-tag and phospho-PKC-substrates antibody was performed in parallel to control the success of the kinase reaction and of the Myc-PKCα pull down.

### Label-free quantitation mass spectrometry and data analysis

Proteins were digested with trypsin, and peptides extracted according to the protocols established by the Proteomics STP lab at the Francis Crick Institute. Peptides were separated on a 50 cm, 75 µm I.D. EasySpray column over a 120 min gradient and eluted directly into an Orbitrap Fusion Lumos mass spectrometer. Xcalibur software was used to control the data acquisition. The instrument ran in Data Dependent Top Speed mode with cycle time of 3 s. The most abundant peptides with charge states 2-4 were selected for tandem mass spectrometry. Data processing was performed by the MaxQuant bioinformatics suite using the LFQ algorithm and “match between runs” option. The Homo sapiens protein database was searched. Any peptide identified from the control samples was removed from analysis. Data was plotted using custom R scripts. Known PKCα interaction data was curated from the STRING and BioGRID databases. Further data analysis and presentation were performed with the Perseus software.

### STRING analysis

STRING analysis (19) was conducted on proteins recovered in PKCα immunoprecipitates. Any proteins recovered in control precipitates were excluded, as were those only recovered in a single sample. For analysis of proteins preferentially associating with the D463H mutant, proteins showing greater than a 0.5 log FC enrichment in the D463H samples over the WT samples were considered hits. This list also includes all D463H exclusive binders. STRING analysis was set at varying levels of confidence with only direct binders included in the query list (no shell); text mining, experiments, databases, gene fusion and co-occurrence are included as potential interaction sources.

### Cell viability assays

U87MG parental and stable transfected U87MG WT-PKCα, D463H, and D463N cells were plated in 96-well plates in 100 µl of complete DMEM with a minimum of five technical replicates. 24 hours later cells were treated with either JQ1 or AZD5351 in a final concentration of 0.1% DMSO (vehicle). A no growth control well was also treated with sodium azide (0.1%). Cells were grown for a further 72 hours prior to the addition of 0.5 mg/ml MTT for 3 hours. Formazan crystals were solubilised in DMSO, and absorbance was measured at 570 nm using a colorimetric plate reader (BMG Spectrostar). Cell viability was calculated relative to vehicle and azide controls. All experiments were conducted as a minimum of three biological replicates. For curve fitting and IC_50_ calculations, [inhibitor] vs response analysis (Non-linear fit, variable slope (four parameters) was conducted using Prism software.

### In silico mutant predictions

AlphaFold 3 was used to predict the structure of full-length PKCα WT, D463H or D463N, in the presence of 1x ATP, 2x Mg^2+^ and 4x Zn^2+^. Twenty seeds (1-20) were used, generating a total of 100 models per protein construct. To classify predictions into PS-in or PS-out conformers, the PAE matrix was used. The mean score of a region 1 with X between [0, 20], Y between [350, 650] (PS-kinase domain interaction) was compared to a region 2 with X between [200, 250], Y between [350, 650] (C2-kinase domain interaction). If the mean error score of region 1<2, the observed conformation was considered PS-in, whereas if the mean score of region 1>2, the observed conformation was considered PS-out. To predict the effect of Asp^463^ substitutions on protein stability DDMut (58) was used with the top-scoring full-length PKC WT model as input.

### Statistical analysis

Statistical analysis of data was performed using t-tests where comparisons are made for groups where there is equal variance. Where comparisons were made relative to controls, we used a one-way ANOVA. Where comparisons were made for sensitivity of cell lines to BET inhibitors, significance was assessed using two-way ANOVA with Tukey’s multiple comparisons. *P*<0.05 was statistically significant. A minimum of *n* = 3 independent experiments was used, as indicated in the figure legends.

## Supplementary Material

fig. s1

## Data Availability

Proteomics data is available at data deposited at PRIDE accession number PXD049959 and RNAseq data is available at the GEO repository accession number GSE269531. Custom R scripts used to analyse and plot data are publicly available at https://github.com/jackchenry/PKCa_Calleja-et-al. All other data required to evaluate the conclusions of the paper are presented in the text, figures, legends, and supplementary materials. Materials are available through the corresponding authors.

## One Sentence Summary

A PKCα catalytic inactivating mutation confers gain-of-function properties and a paradigm shift in kinase actions.
