# Supplementary material for: The chordoid glioma-associated PRKCA D463H mutation is a kinase inactive, gain-of-function allele that induces chondrosarcoma in mice
Source: Sci Signal. Author manuscript; Available in PMC 2025 Nov 25. (PMC7618404; doi:10.1126/scisignal.adr0235)
Supplement: fig. s1 [file EMS210955-supplement-fig__s1.docx]

**Supplementary Materials**

**Supplementary Figures**

**
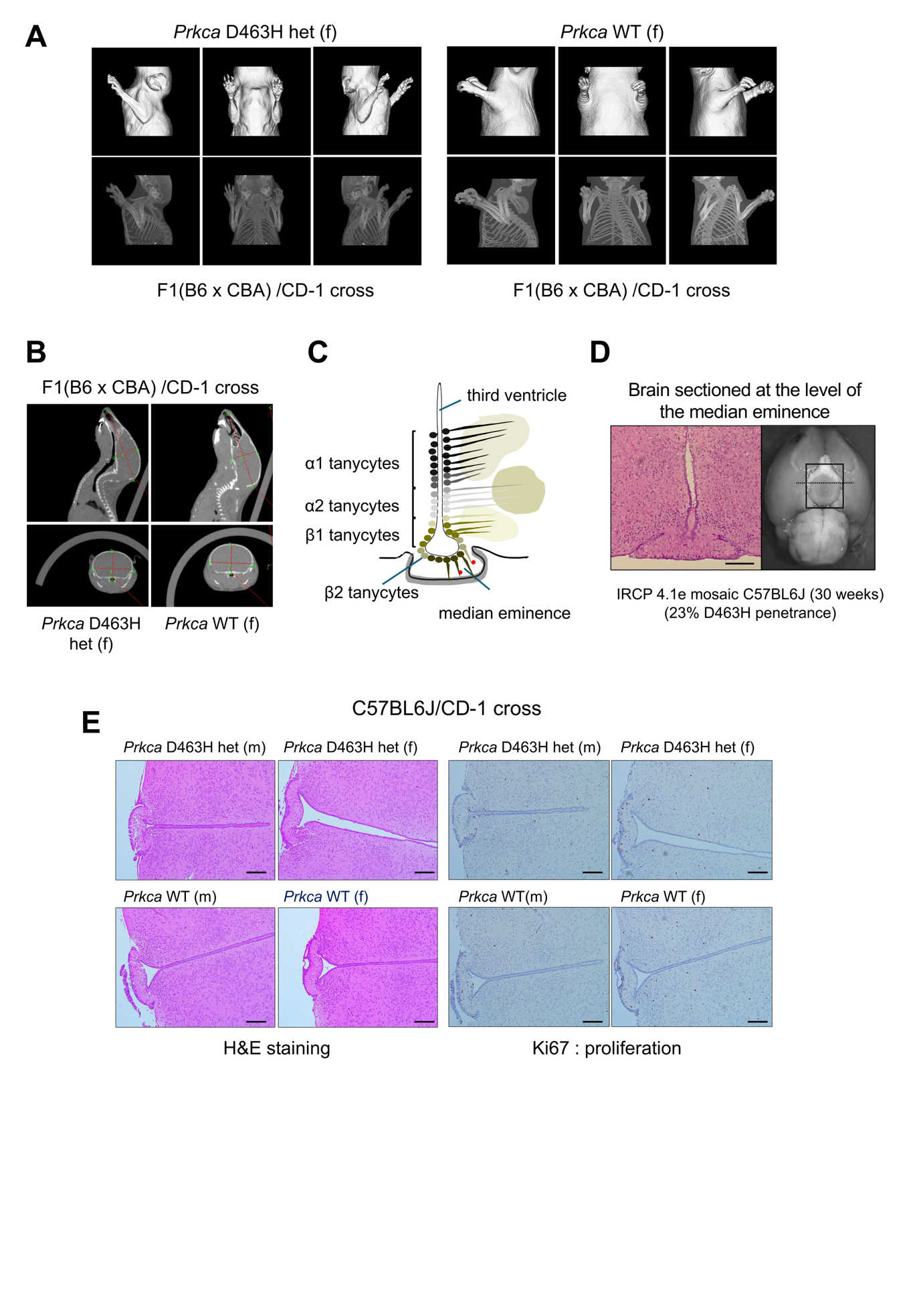
**

**Fig. S1. Forelimbs and cranial analysis in heterozygous PRKCA-D463H knockin mice.** (**A**) CT-scan of the forelimbs of a D463H mouse compared to WT; 2 animals were analysed. (**B**) CT-scan of a head of a D463H Het mouse compared to WT; 2 animals were analysed. (**C**) Schematic of the mouse brain median eminence and the different types of cells. (**D**) H&E on a 23% D463H mosaic brain’s median eminence of the one surviving 30 week old mouse (scale bar 200 µm). (**E**) H&E and Ki65 median eminence staining of D463H het (male and female 2 week old) mice (scale bar 200 µm); n=3.


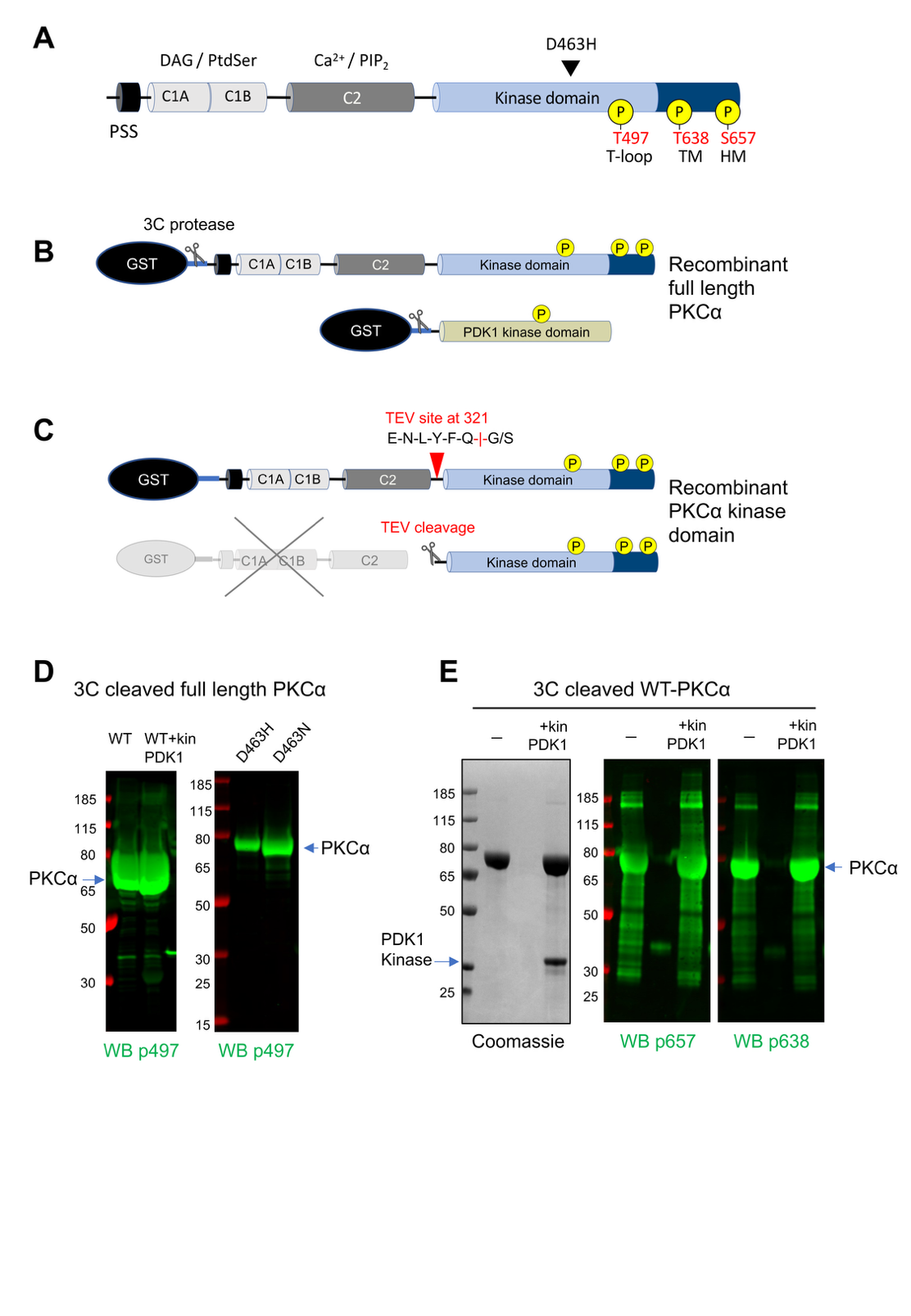


**Fig. S2. Recombinant PKCα constructs expression and phosphorylation.** (**A**) representation of the PKCα domains (C1A, C1B, C2) and phosphorylated activation sites in the kinase and C-terminal domains (T-loop or activation loop; TM: turn motif; HM: hydrophobic motif). The pseudo substrate site (PSS) is shown at the N-terminus of the sequence. The D463H mutation is shown on the kinase domain. (**B**) and (**C**) representation of the strategies used to produce full length and kinase domain recombinant PKCα. The full length protein is cleaved with TEV protease at a TEV cleavage site introduced at the residue 321 to yield the kinase domain. (**D**) Activation loop phosphorylation (pT497) of full length WT-PKCα and D463H and D463N mutants expressed in the presence or absence of co-expressed PDK1 kinase domain (n=3). (**E**) Turn motif (pT638 (TM)) and hydrophobic motif (pS657 (HM)) phosphorylation of full length WT-PKCα expressed in the presence or absence of the PDK1 kinase domain. Phosphorylation was detected by western blot and the expression of the protein visualized by Coomassie blue staining (n=3).


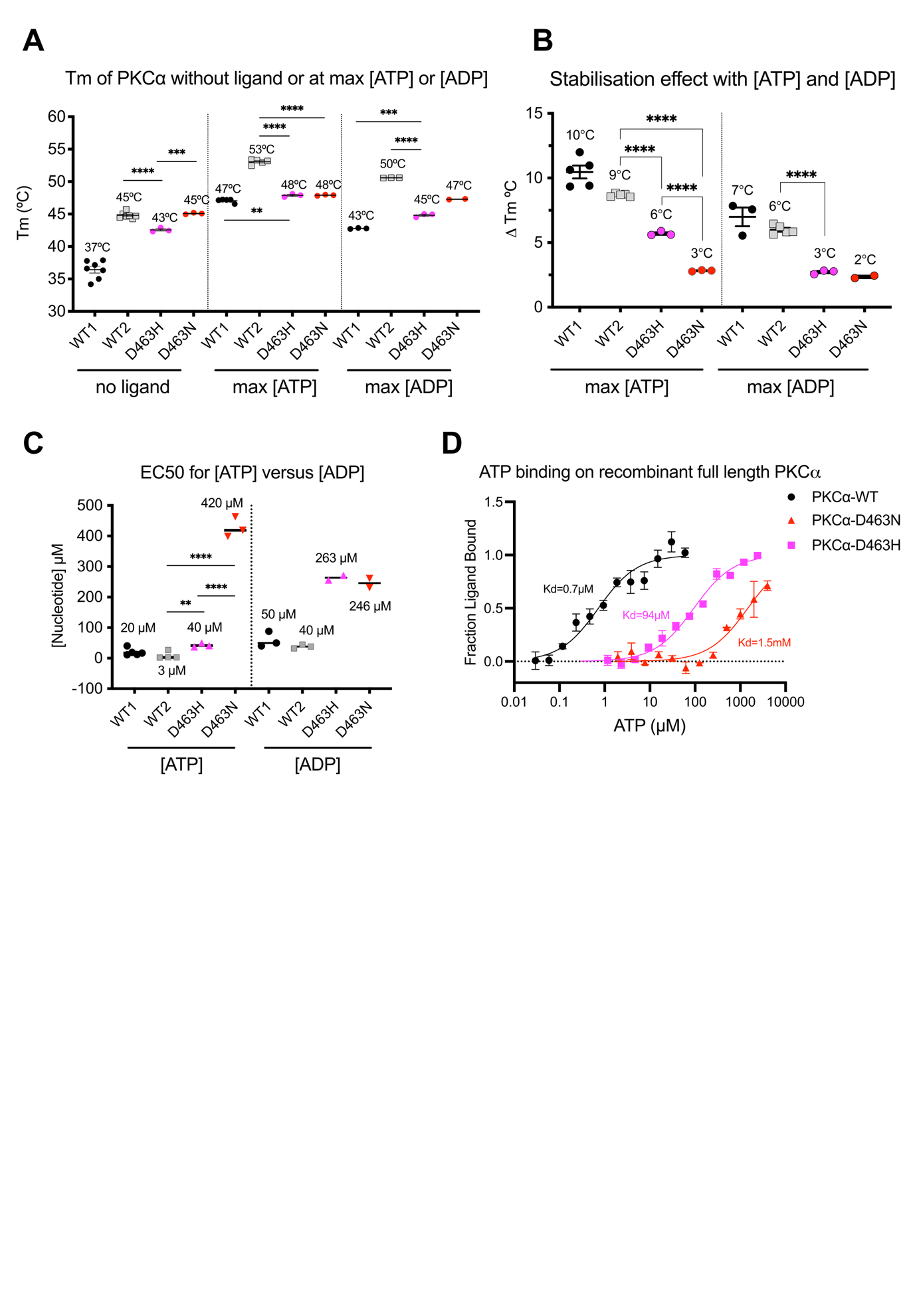
 **Fig. S3. Thermal shift assay and microscale thermophoresis.** Recombinant WT-PKCα and mutants were mixed with ATP or ADP in presence of a fluorescent dye. (**A**) melting temperatures (Tm) determined by the fitting the melting curves of recombinant WT-PKCα and mutants with different nucleotides present where *n* = 3 to 7 (as indicated by individual data points). (**B**) The variation of the melting temperature (Tm) prior to (Apo) or in the presence of nucleotides (ATP or ADP) allows the calculation of the ∆Tm. An unpaired t-test was used to determine the significance of the different changes for *n* = 3 to 5 (as indicated by individual data points). (**C**) Apparent affinities (EC_50_) calculated from the increase in Tm, of WT-PKCα versus D463H and D463N mutants for ATP or ADP. In all the TSA experiments the significance was assessed using an unpaired t-test *P*<0.01 (**), *P*<0.001(***), *P*<0.0001 (****). (**D**) ATP binding affinities (Kd) were determined on full-length recombinant PKCαWT and mutants by microscale thermophoresis (*n*=3).


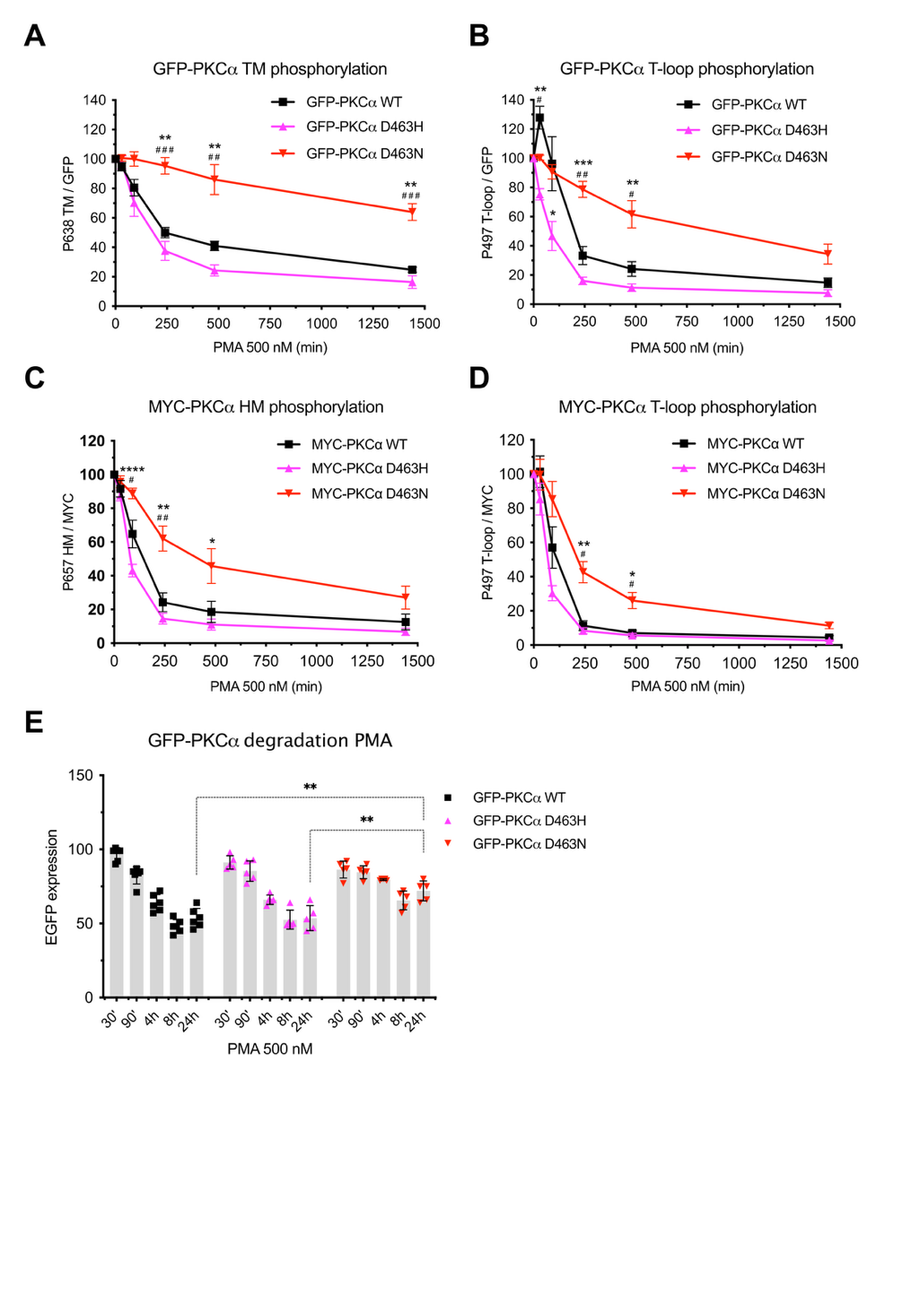


F**ig. S4. Similar regulation of WT-PKCα and D463H mutant constructs upon PMA treatment.** (**A-D**), GFP-PKCα (graphs A and B) and Myc-PKCα (graphs C and D) WT and mutants were transiently expressed in U87MG cells. The cells were treated with 500 nM PMA for up to 24 hours. The T-loop, hydrophobic motif or Turn motif (TM) phosphorylation is shown for WT-PKCα and mutant constructs. (**A**) Comparison of the TM phosphorylation of D463N vs D463H (*P* values*) and D463N vs WT (*P* values^#^); *n* = 3. (**B**) Comparison of the T-loop phosphorylation was done for D463N vs D463H (*P* values*) and D463N vs WT (*P* values^#^); *n* = 3. (**C**) Analysis was performed to compare the HM phosphorylation of D463N vs D463H (*P* values*) and D463N vs WT (*P* values^#^); *n* = 4. (**D**) Comparison of the T-loop phosphorylation of D463N vs D463H (*P* values*) and D463N vs WT (*P* value^#^); *n* = 3. (**E**) The graph shows the variation of GFP-tagged PKCαWT, D463H and D463N mutants expression upon prolonged PMA treatment (*n* = 5). In all experiments the confidence was assessed using an unpaired t-test *P*<0.05 (*), *P*<0.01 (**), *P*<0.001(***), *P*<0.0001 (****).


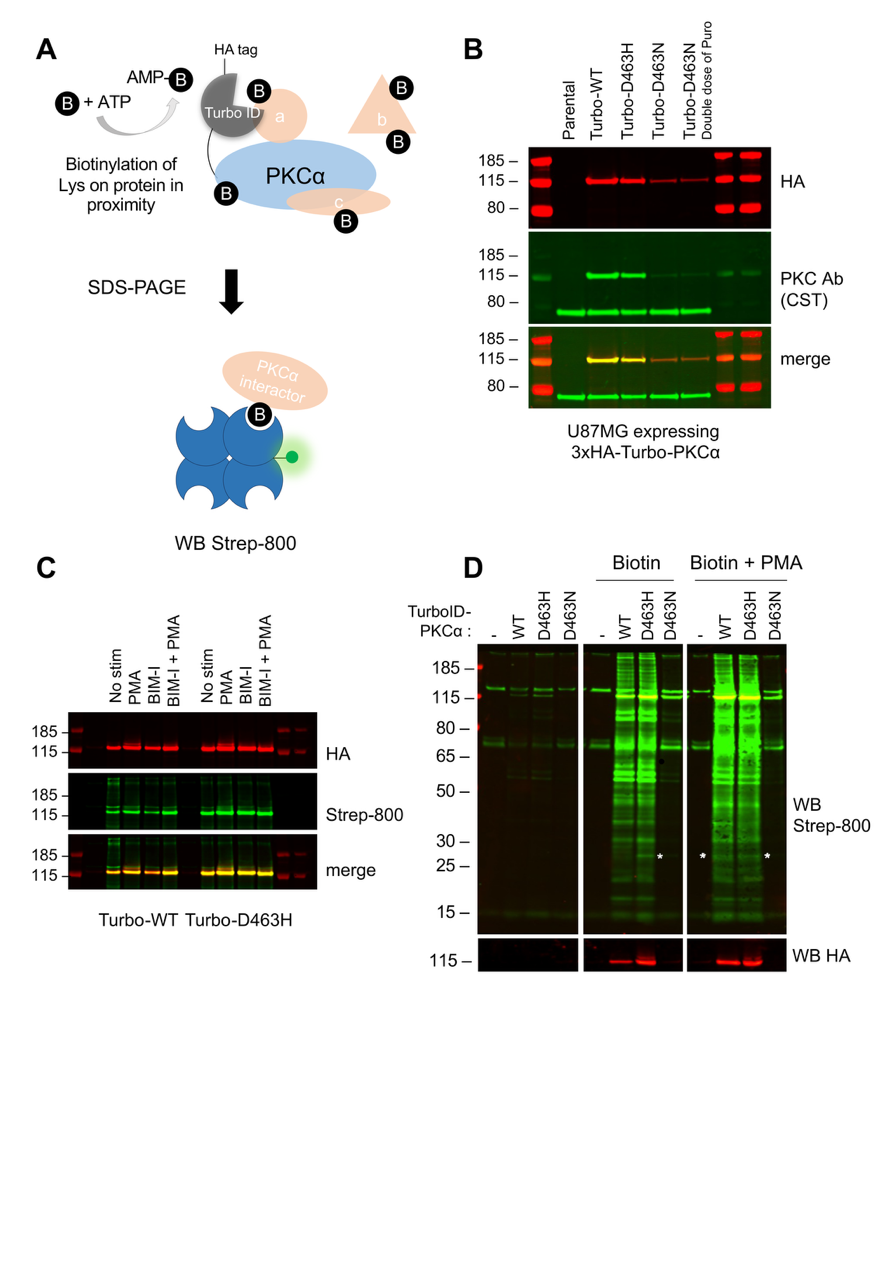


**Fig. S5. TurboID-PKCα biotinylation of PKCα and mutants.**

(**A**) Schematic principle of TurboID-tagged PKCα biotinylation (black circle, labelled with ‘B’) of associated proteins (light pink) and PKCα itself and recognition of the biotinylated proteins with streptavidin labelled with IRDye800. (**B**) Stable expression of 3xHA-TurboID-PKCα WT and D463 mutants in U87MG cells detected by western blot with anti HA or anti PKCα antibodies; one of 3 similar experiments. (**C**) Western blot anti HA or Streptavidin following neutravidin pull down of 3xHA-TurboID-PKCα from U87MG stable cell lines. The expression (red) and biotinylation status (green) of 3xHA-TurboID-PKCα WT and mutants is shown upon treatment with PMA or BIM-I; one of 3 similar experiments (**D**) Western blot with anti HA or fluorescently labelled Streptavidin following neutravidin pull down of 3xHA-TurboID-PKCα from U87MG stable cell lines. The neutravidin pull down of biotinylated proteins from cells stably expressing 3xHA-TurboID-PKCα WT and mutant D463H is compared to the pull down of untransfected parental U87MG cells prior to or upon PMA treatment. The expression of PKCα is shown by western blot anti HA. The white stars indicate the protein differently pulled down in D463H conditions compared to WT. One of three such experiments.

**Fig. S6. Structure predictions for WT and mutant forms of PKCα.** (A) Top scoring AlphaFold3 models for PKCα WT, D463H and D463N mutants, models are shown in a rainbow colour scheme on the left and according to the predicted Local Distance Difference Test (pLDDT) score to the right (a scale bar is shown at the bottom of this column). The PAE matrices are shown next to the models. the in/out denotation represents whether the model presents in a PS-in or PS-out conformation (see text). (B) Model classification per conformation (PS-in or PS-out). (C) pTM scores (higher means more reliable) of models observed in a PS-out conformation for WT and mutant PKCα predictions. (D) Median PAE scores (lower means better confidence) for models observed in a PS-out conformation for WT and mutant PKCα predictions. (E)  pTM scores of models observed in a PS-in conformation for WT and mutant PKCα predictions. (F) Median PAE scores for models observed in a PS-in conformation for WT and mutant PKCα predictions. Data in C-F are presented as violin plots and were analysed using one-way ANOVA followed by Tukey’s multiple comparisons test. Statistical significance is indicated by ** p ≤ 0.01, **** p ≤ 0.0001.

**
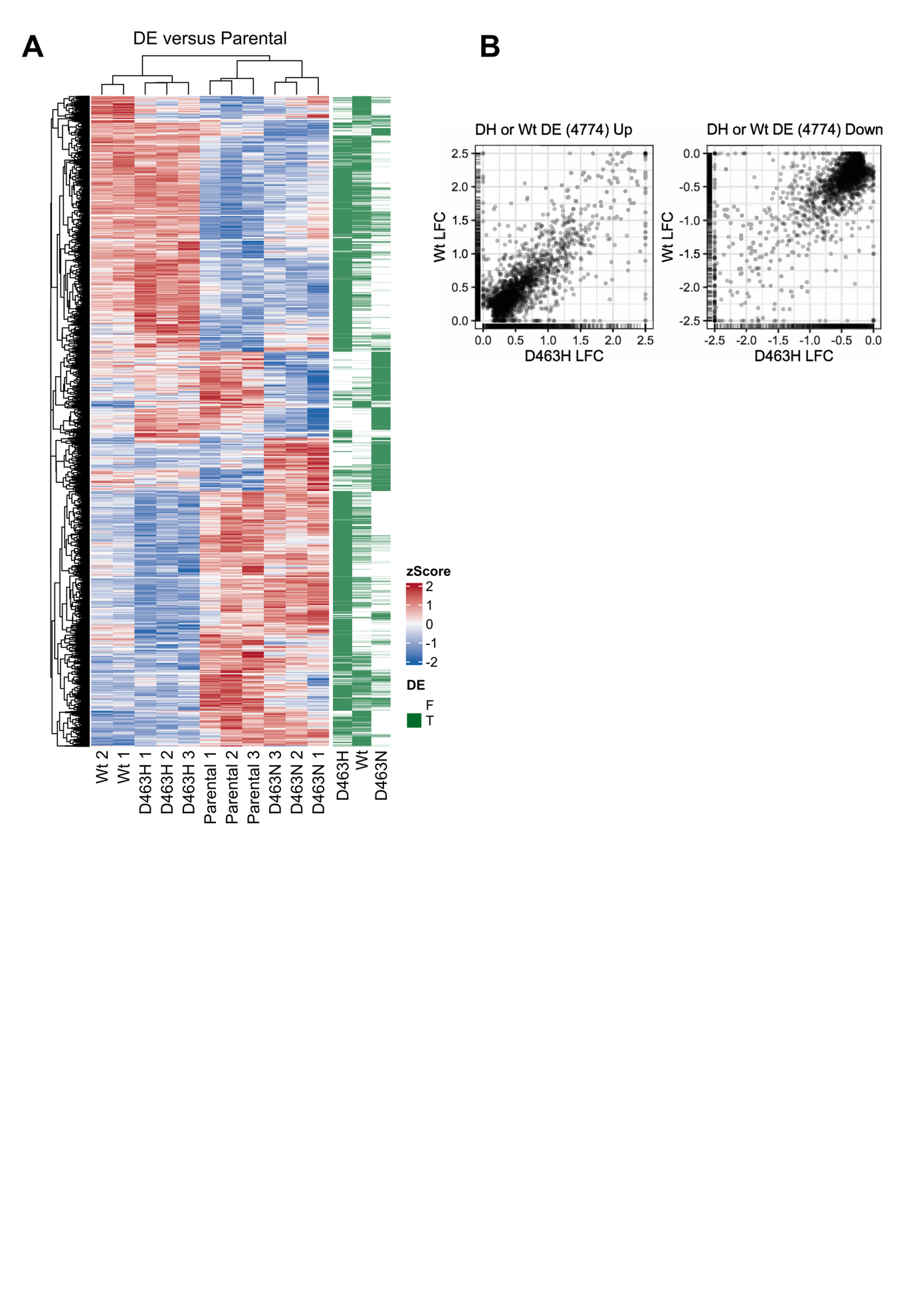
**

**Fig. S7. PKCα D463H is a gain-of-function mutant.** (**A**) Heatmap of genes differentially expressed on overexpression of Myc-tagged WT-PKCα, D463H and D463N compared to parental cells. Tiles are coloured by per-gene z-scores across all samples. Right heatmap annotation identifies differential gene expression for each group compared to parental (replicates as numbered in the figure). (**B**) Scatter plots describing the relationship in log fold change in gene expression between WT-PKCα and D463H mutant for genes differentially expressed in WT-PKCα or D463H stable U87MG cells when compared with parental cell lines.

**
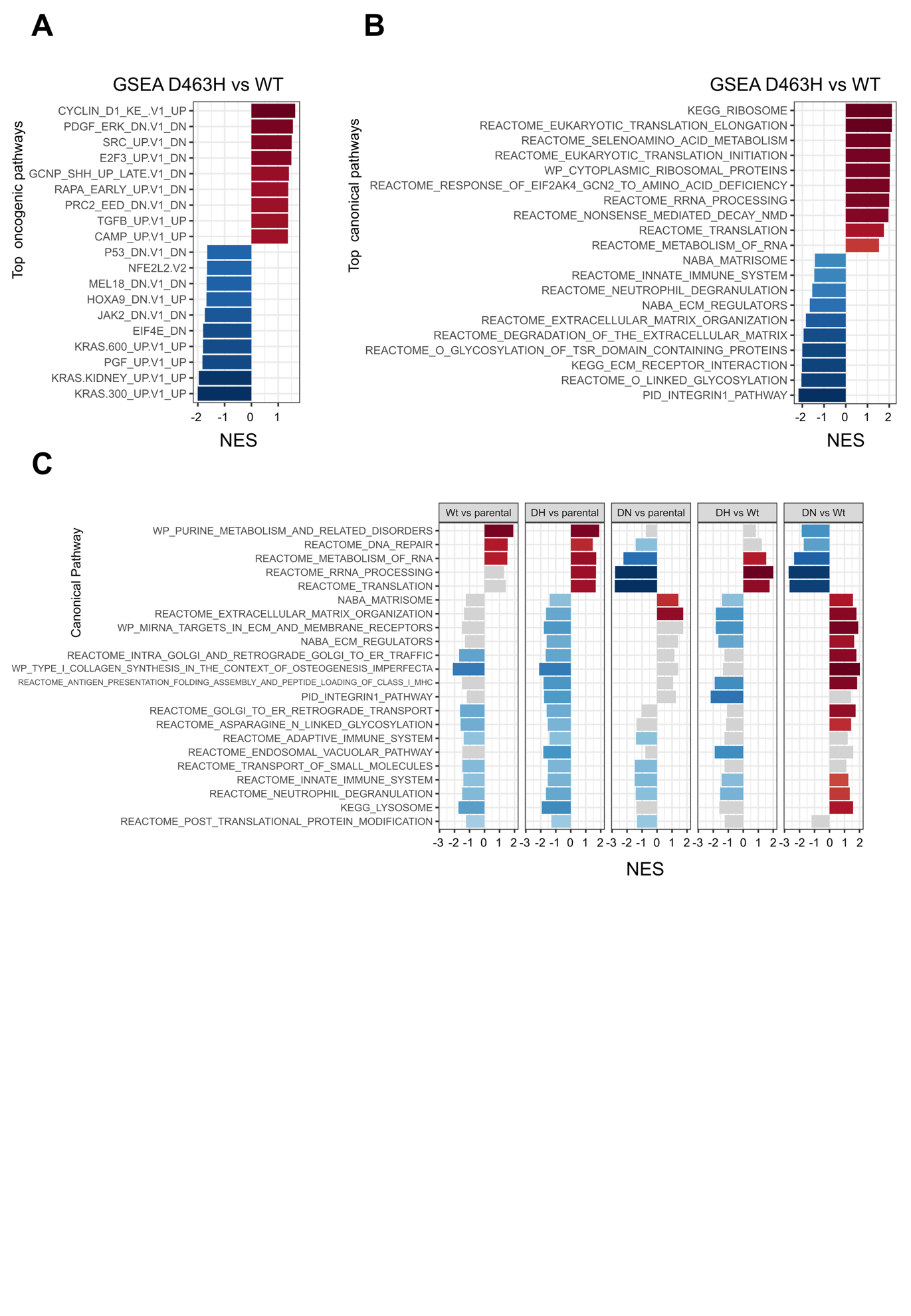
**

**Fig. S8. GSEA analysis of differential oncogenic and canonical pathways between WT-PKCα and D463H.** (**A-C**) Normalised enrichment scores from gene set enrichment analysis for the oncogenic pathways (A), canonical pathways (B, C) collections obtained from the molecular signatures database. Ranks were calculated based on the Wald statistic from differential expression results between D463H and WT-PKCα U87MG cells in A and B and in all differential expression comparisons in C. The top and bottom ten most significantly enriched pathways from the collections are shown in A and B. Gene sets with two or more agreements between the WT-PKCα versus parental, D463H versus parental and D463H versus WT-PKCα samples are shown in C.

**Supplementary Tables**

**Table S1. *Prkca*-D463H mosaic mice obtained from the first round of CRISPR/Cas9 genome editing.** From the first round of CRISPR in C57BL/6J mice, the mosaic male IRCP4.1c (in red) with 22% penetrance of the mutation D463H, survived and was used for timed mating.


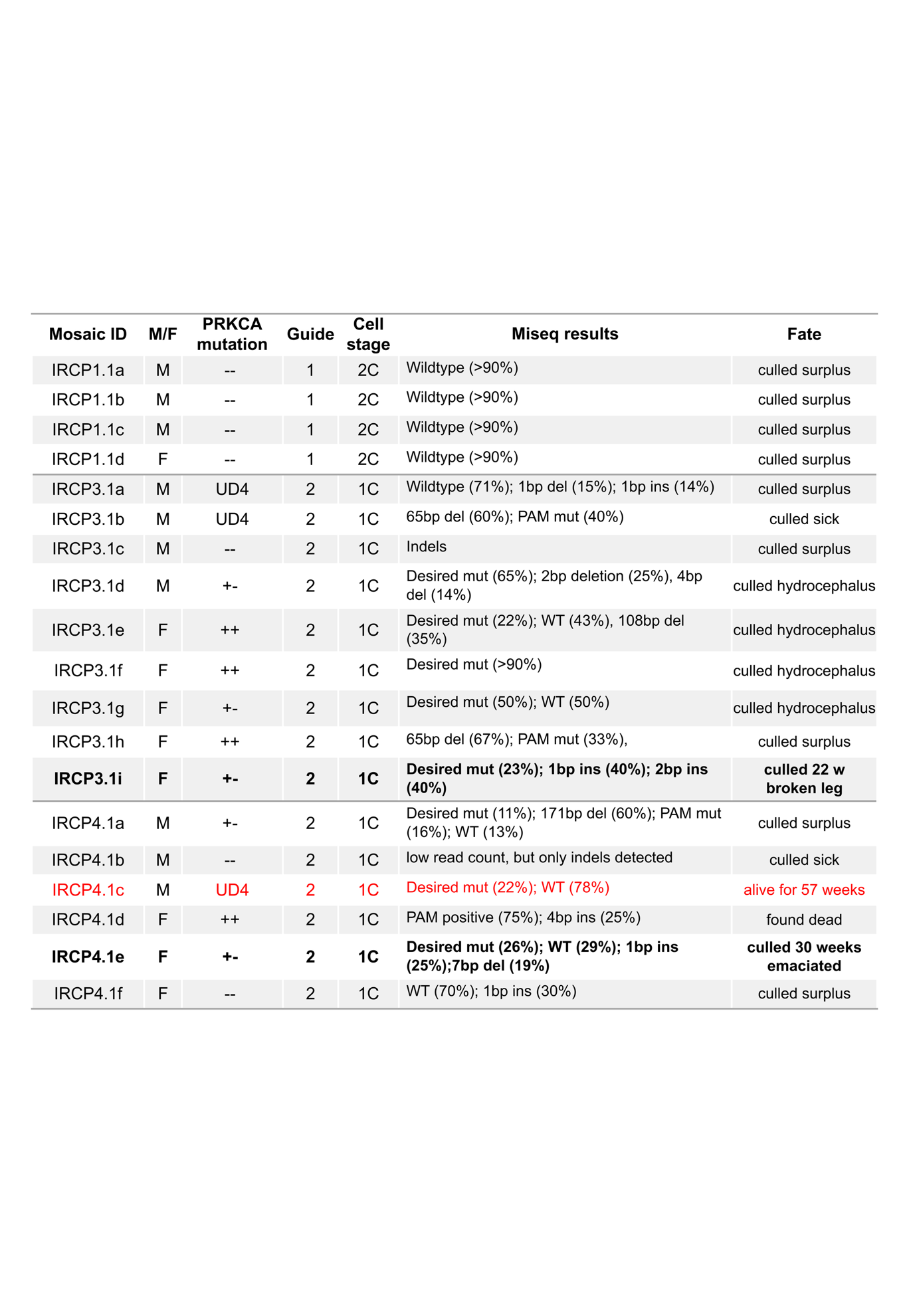


**Table S2. *Prkca* D463H Het mice obtained from IRCP4.1c mosaic mice by timed mating.** Offspring from the timed mating of the male mosaic mouse IRCP4.1c (table S1) with C57BL/6J females. 7 heterozygous mice were identified out of 46 pups and all displayed an adverse phenotype and were culled.


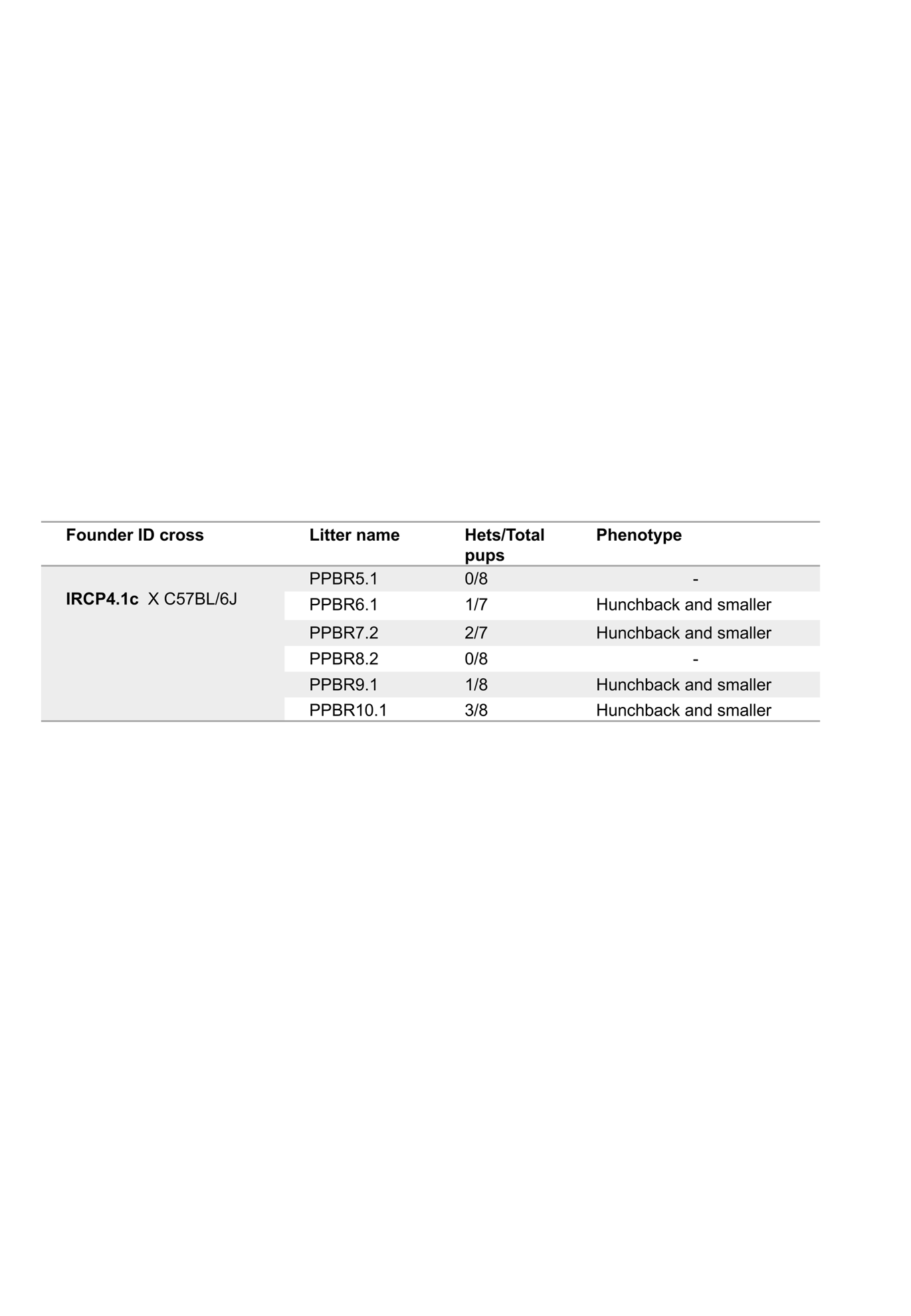


**Table S3. *Prkca* D463H Het mice obtained from IRCP4.1c mosaic mice by IVF of CD-1.** CD-1 female mice were used for IVF with the mosaic male IRCP4.1c (table S1). The 3 born heterozygotes from 4 litters displayed a deleterious phenotype. Only one D463H het male (PPBY4.1c) survived up to 11 weeks.


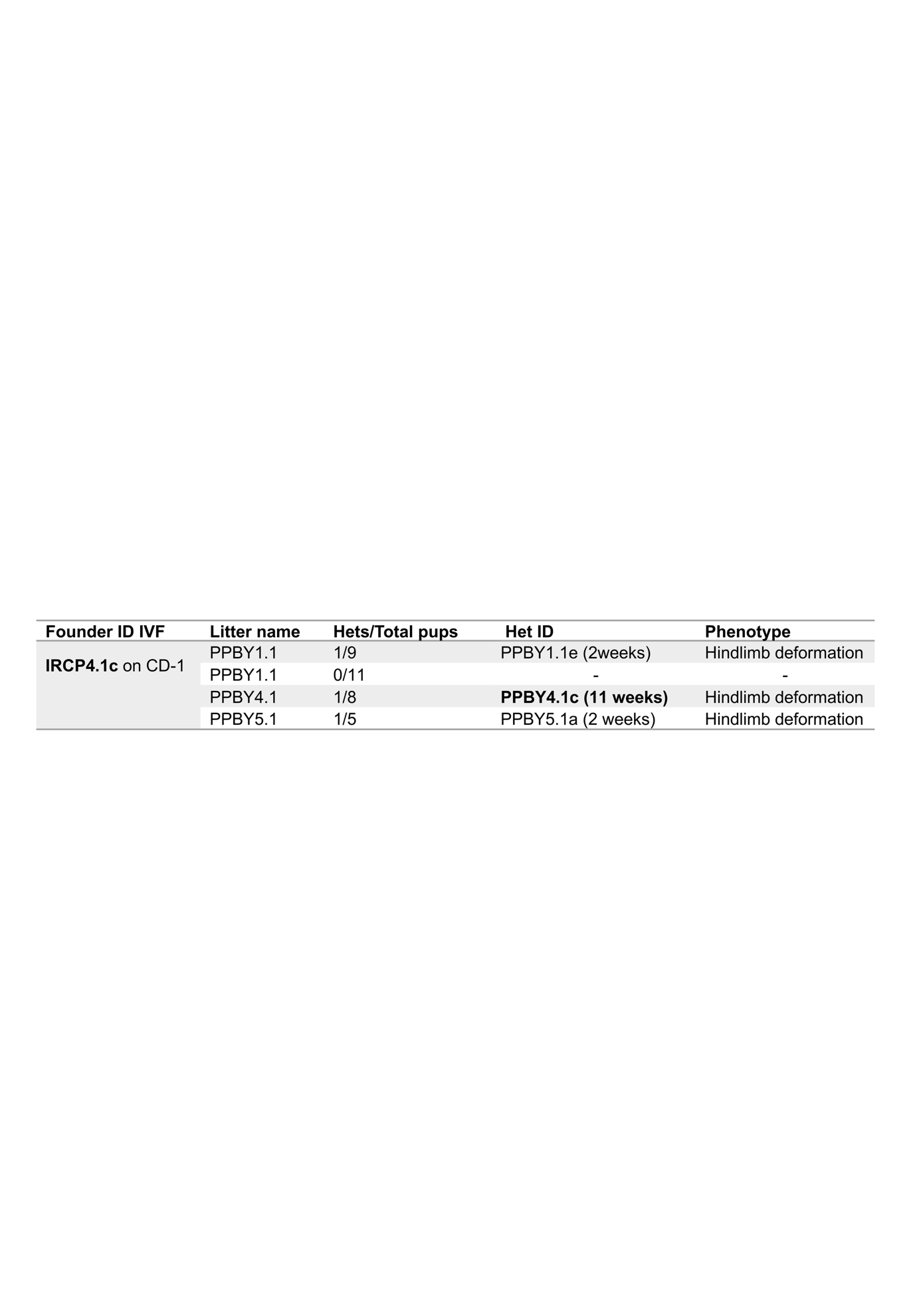


**Table S4. *Prkca* D463H cross for Het mice with PPBY4.1c in trio with CD-1.** The D463H Het male PPBY4.1c was placed in trio with CD-1 female mice and produced 4 hets who did not survive later than 9 weeks, all displaying a harmful phenotype of hindlimb deformation.


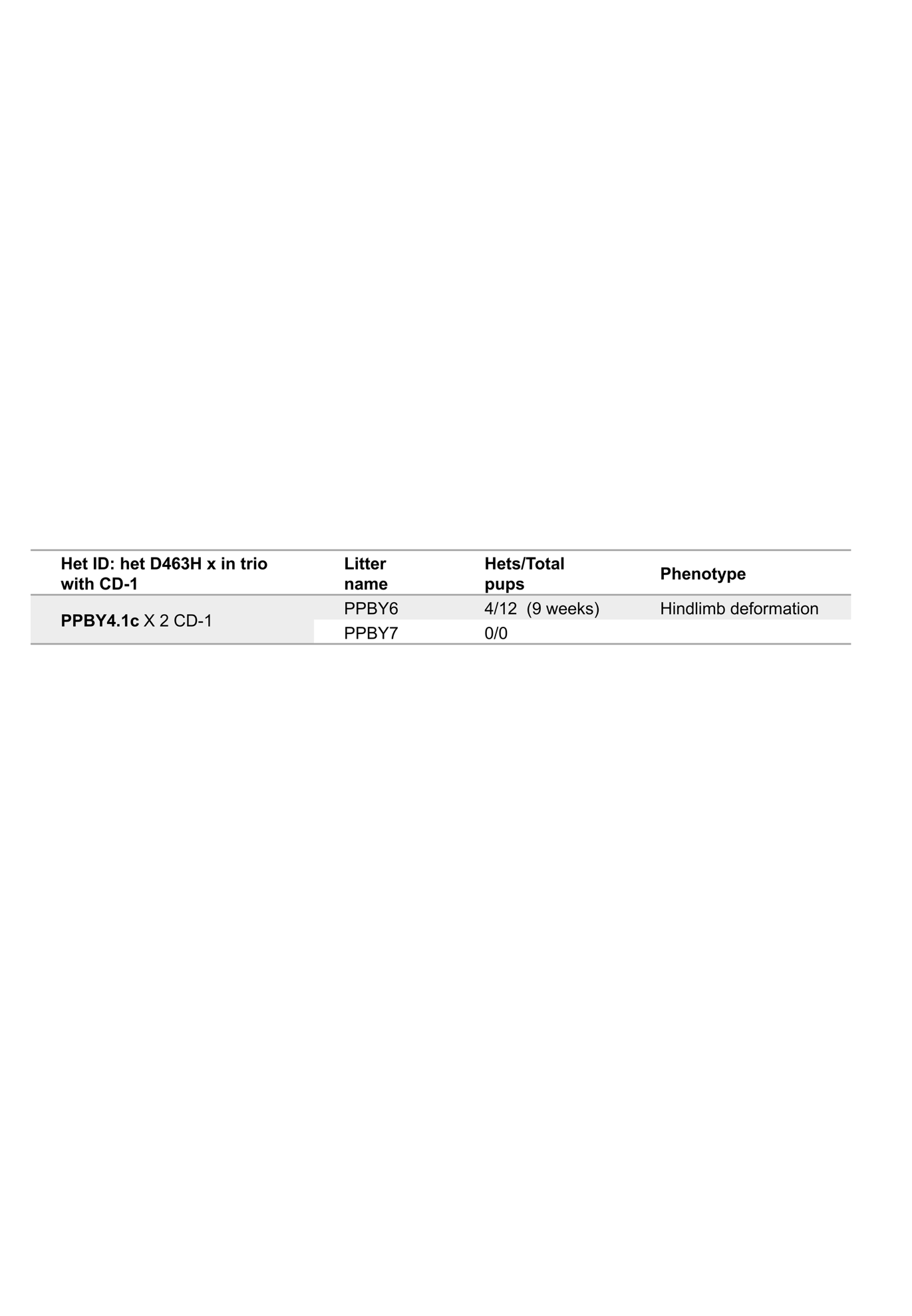


**Table S5. *Prkca* D463H Het mice obtained from 2 mosaic mice from second round of CRISPR/Cas9 genome editing.** Mosaic mice were obtained from a second round of CRISPR on a C57BL/6J background or on an F1(B6 x CBA) background (IRCP8.1e and IRCP20.1b respectively). Both mosaic males were crossed with two CD-1 females. The 21 *Prkca* D463H heterozygotes produced from both male’s backgrounds were sickly and displayed a deformation of the hindlimbs.

**
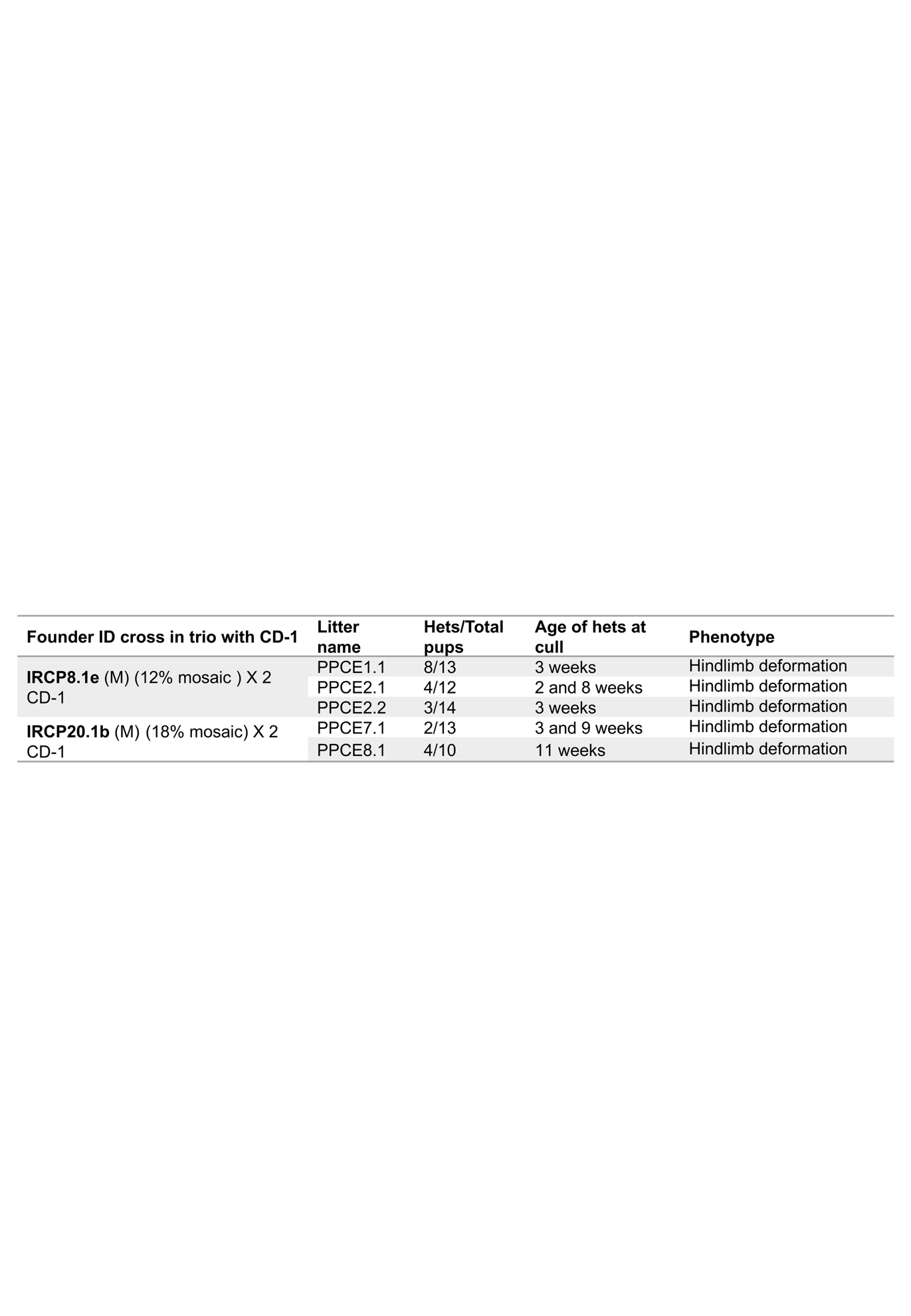
**

**Table S6. *Prkca*-D463N control mosaic mice obtained from CRISPR/Cas9 genome editing.** The same guide RNAs used for the D463H mutation but with a modified repair template was used to produce mosaic mice harbouring the control mutation D463N. 16 mosaic C57BL/6J mice were obtained with the mutation D463N, and 3 of them (IRCY3.1h, IRCY6.1b and IRCY6.1L) were bred for germline transmission.

**
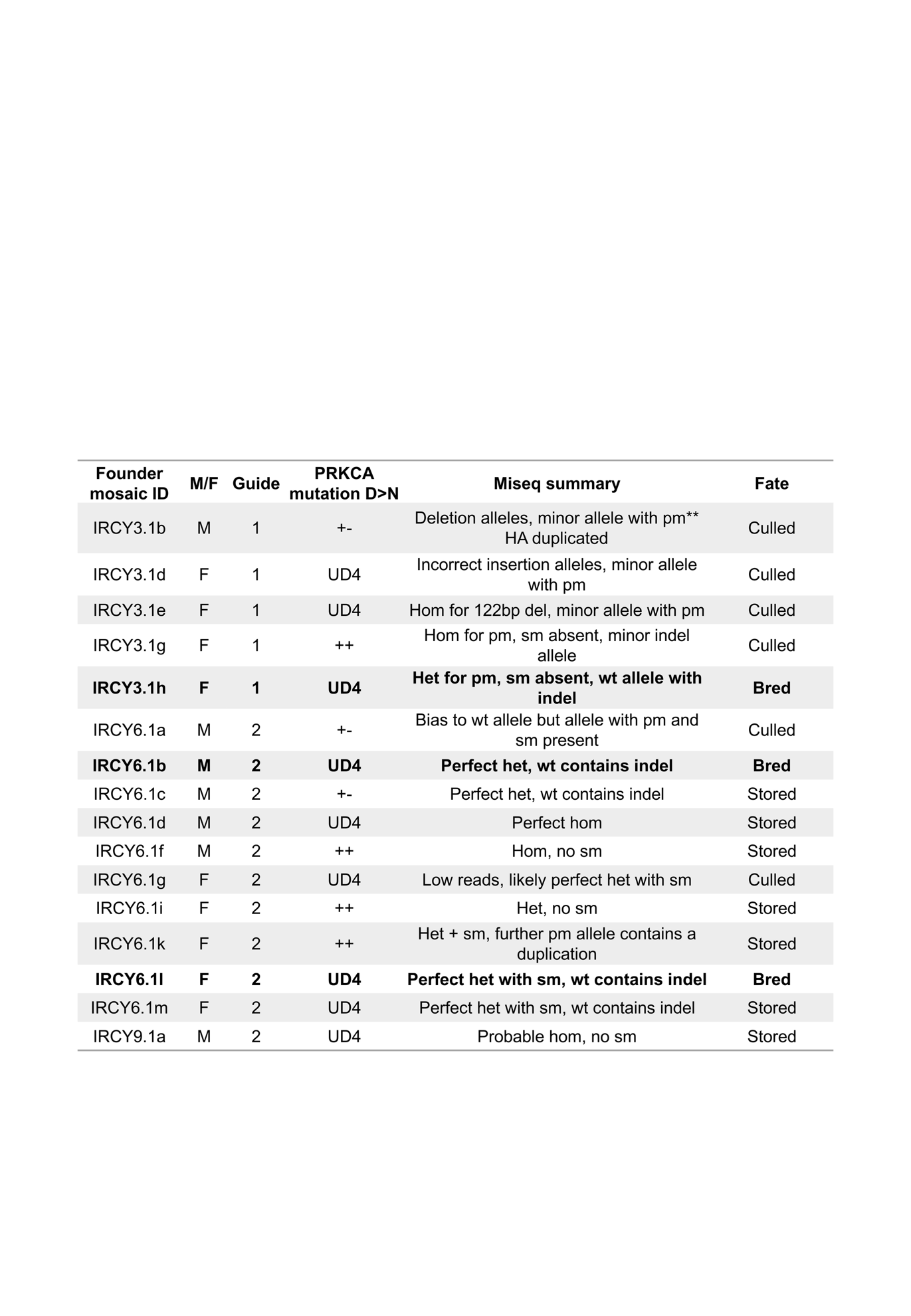
**

**Table S7. *Prkca* D463N control Het mice obtained from three mosaic mice from CRISPR/Cas9 genome editing.** Three mosaic D463N mice (IRCY3.1h, 6.1b and 6.1l) were crossed with C57BL/6J mice to obtain 15 *Prkca* D463N heterozygous mice.


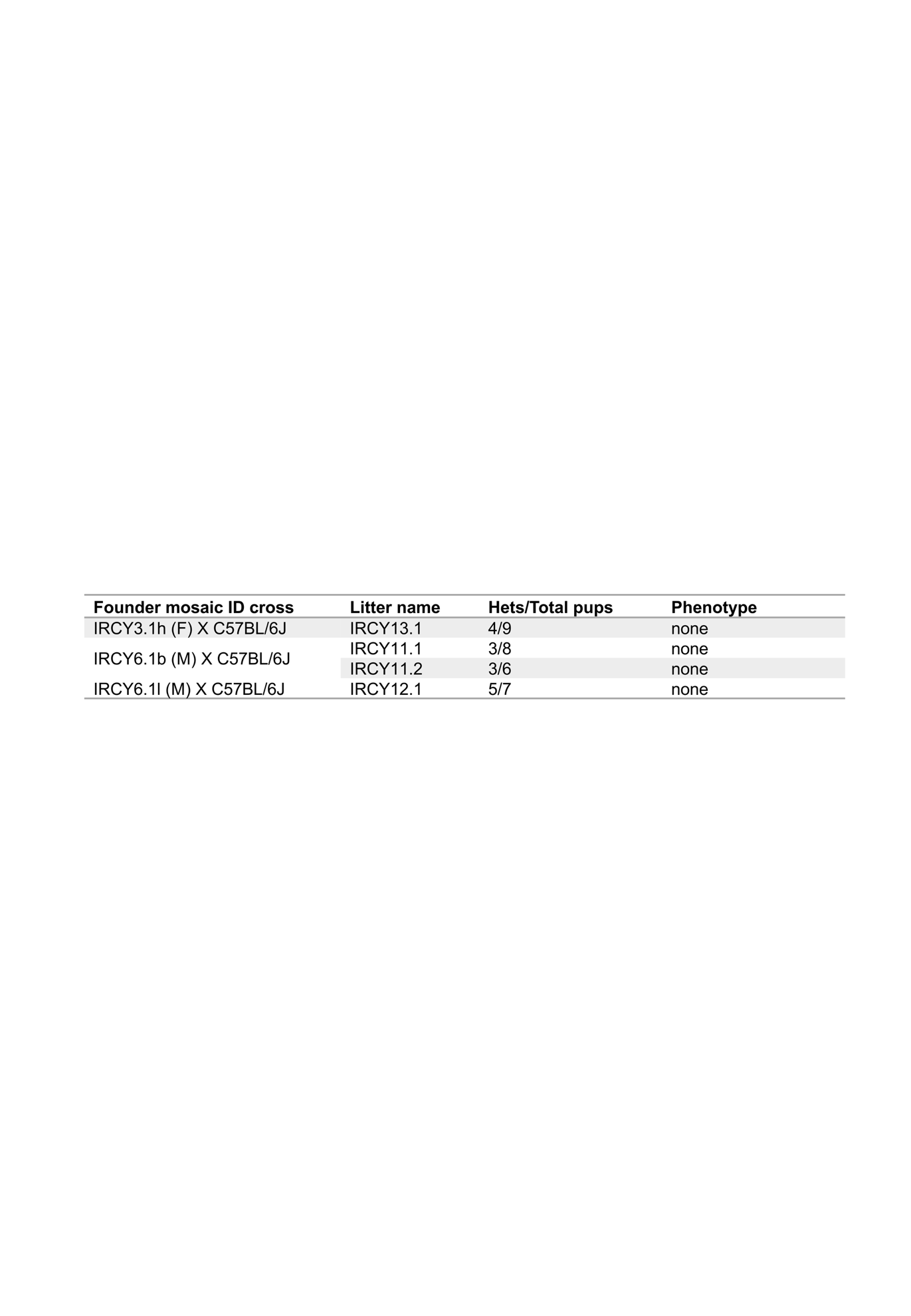


**Table S8. *Prkca* D463N control second round of cross for Het mice.** *Prkca* D463N het offspring from the litters IRCY11, IRCY12 or IRCY13 (see table S7) were crossed with either C57BL/6J, F1(B6 x CBA) or CD-1 mice to obtain a second round of *Prkca* D463N heterozygotes (litters PPBA, PPCB and PPBZ).

**
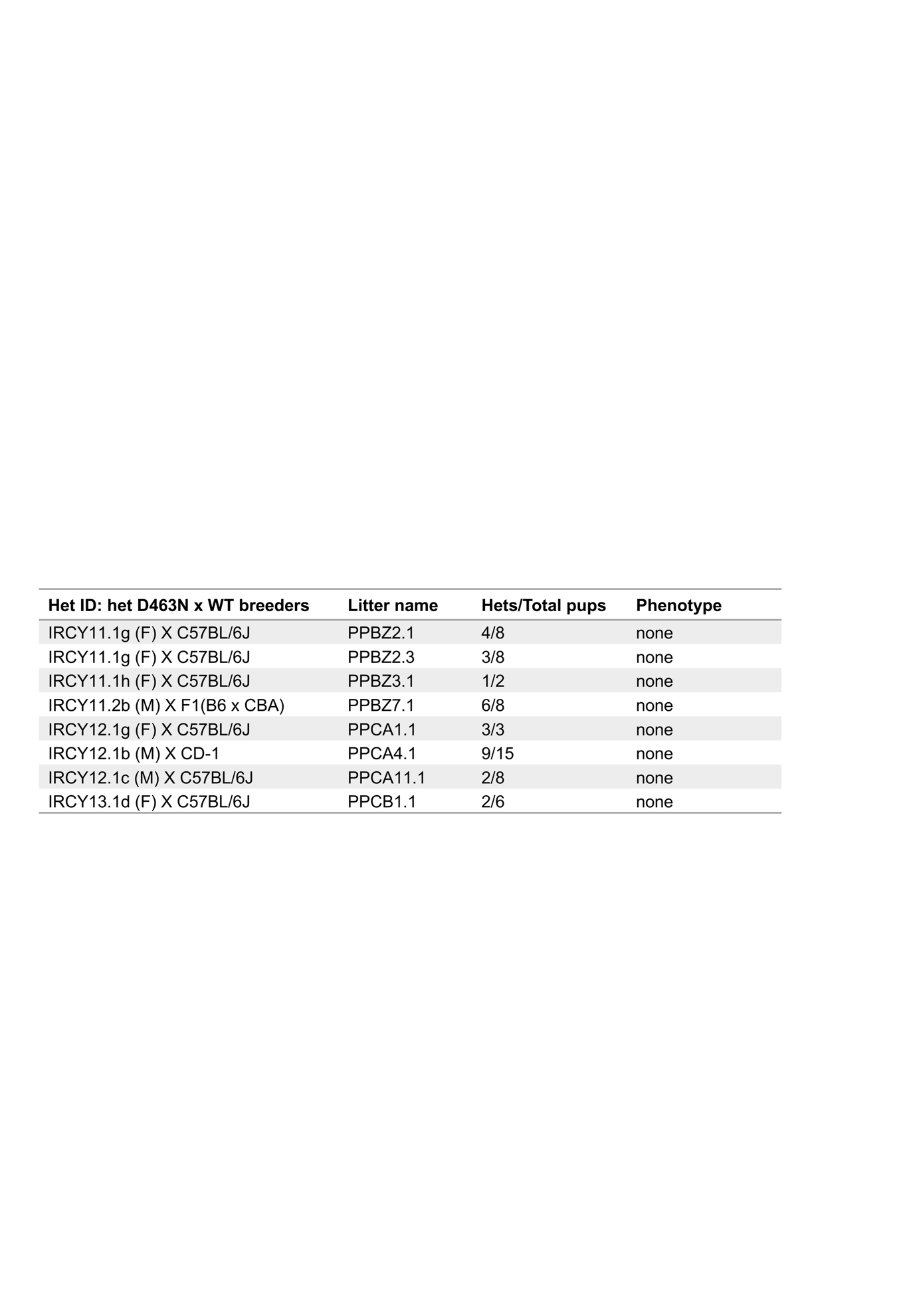
**

**Table S9. *Prkca* D463N control cross for Homozygous mice.** *Prkca* D463N het mice from 3 different litters (see table S7) were crossed. From two different crosses, 12 homozygotes, 14 heterozygotes and 6 WT pups were generated (litters PPBZ8, PPCA12 and PPCB12).


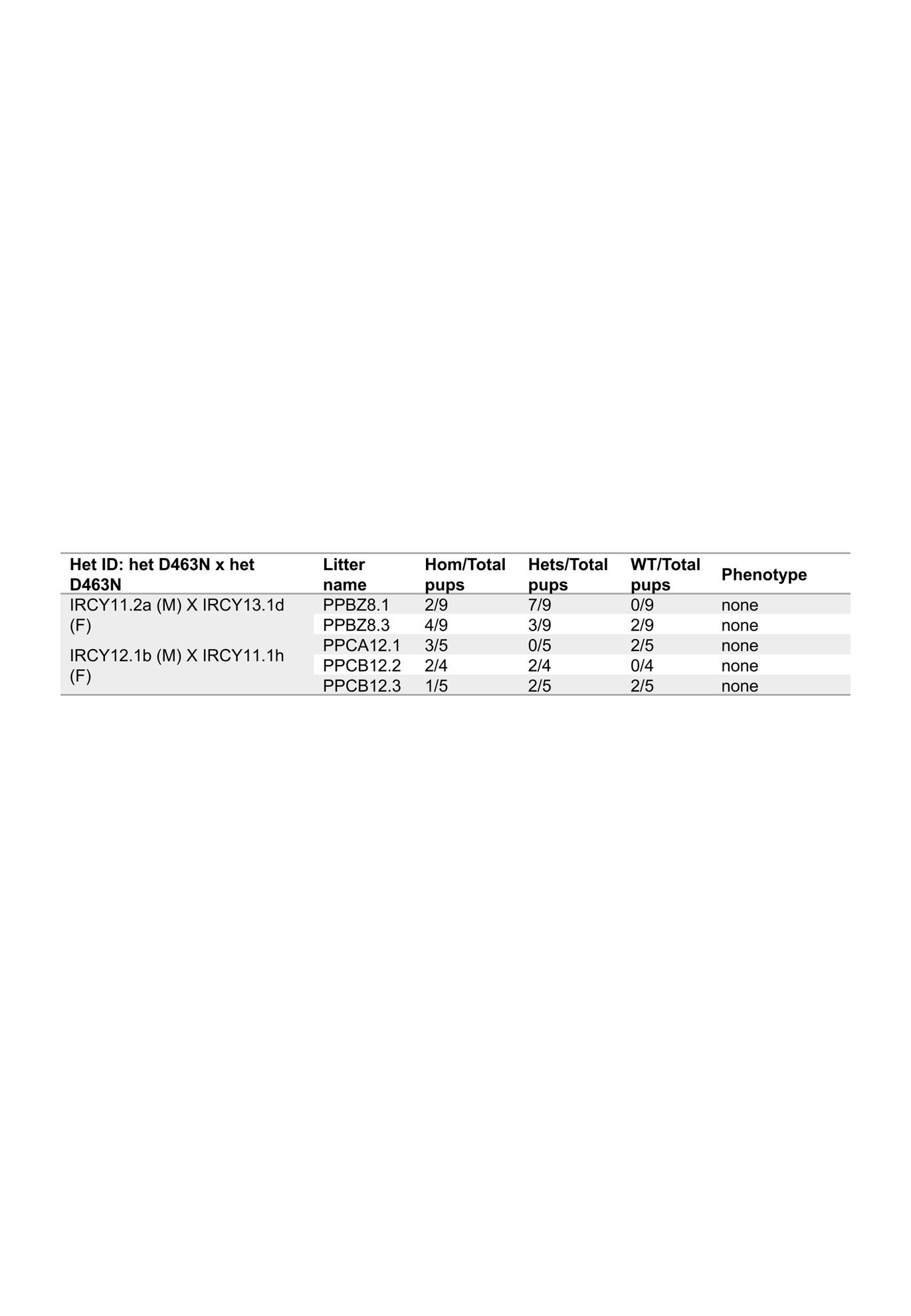


**Table S10. Guide RNAs for knock-in studies.**

**
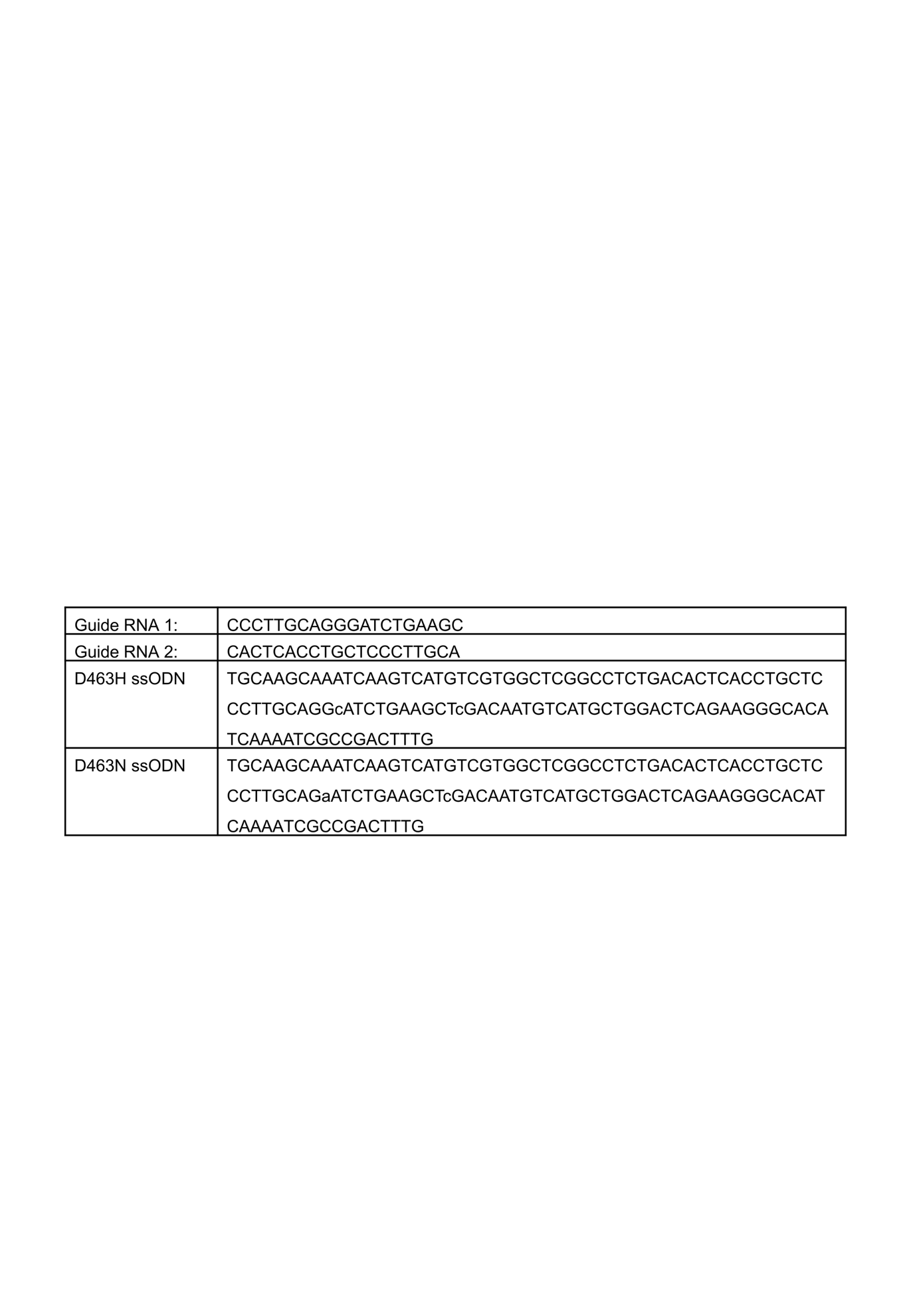
**

**Table S11. Oligonucleotide sequences for construct cloning.**

**
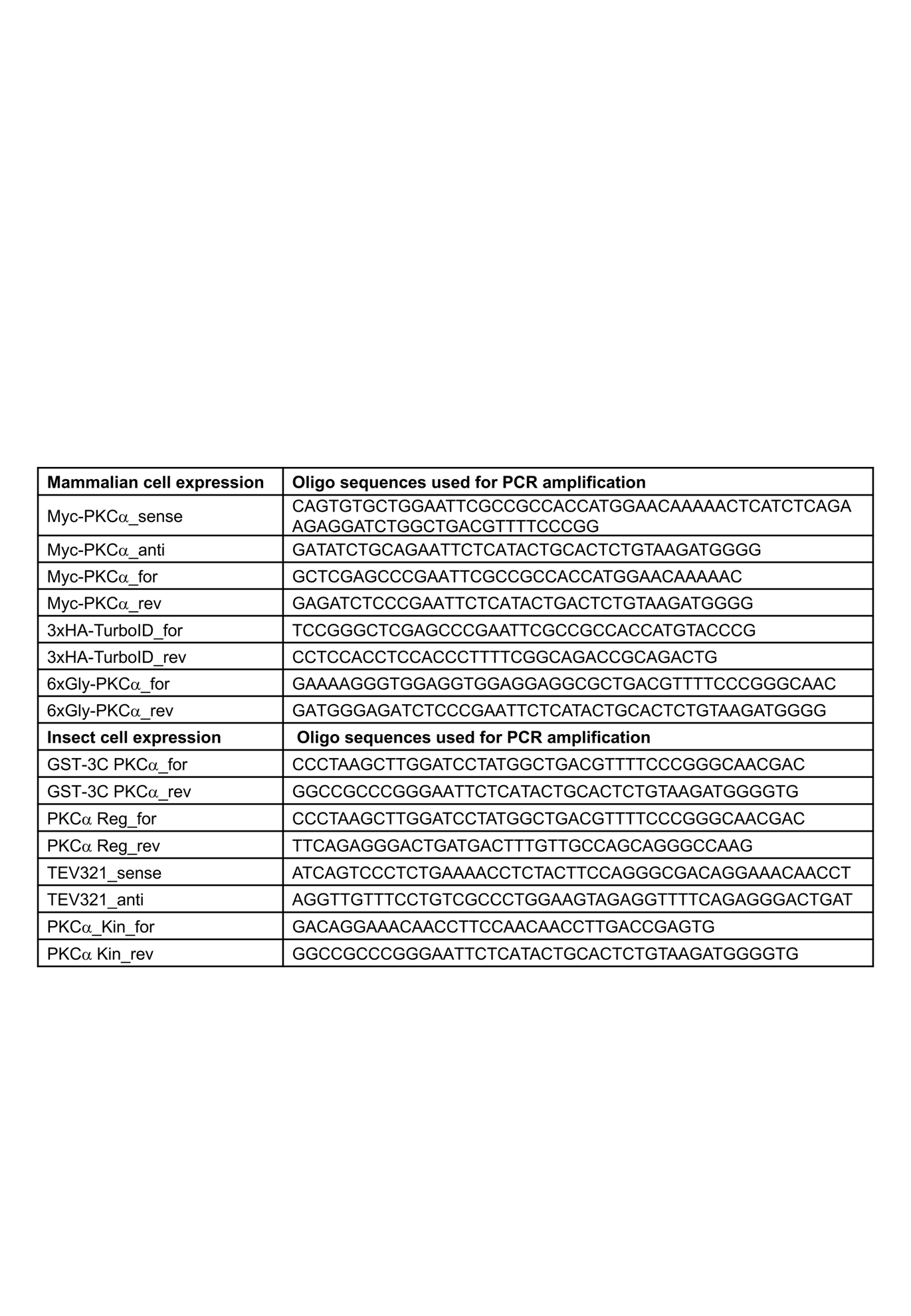
**

**Table S12. Exclusive PKCα mutant D463H interactors.** Proteins exclusively co-immunoprecipitated with MYC-tagged D463H-PKCα. Only proteins identified in a minimum of 2 out of 3 D463H- PKCα replicate precipitates, which did not co-precipitate in any control, WT or D463N sample, are presented.


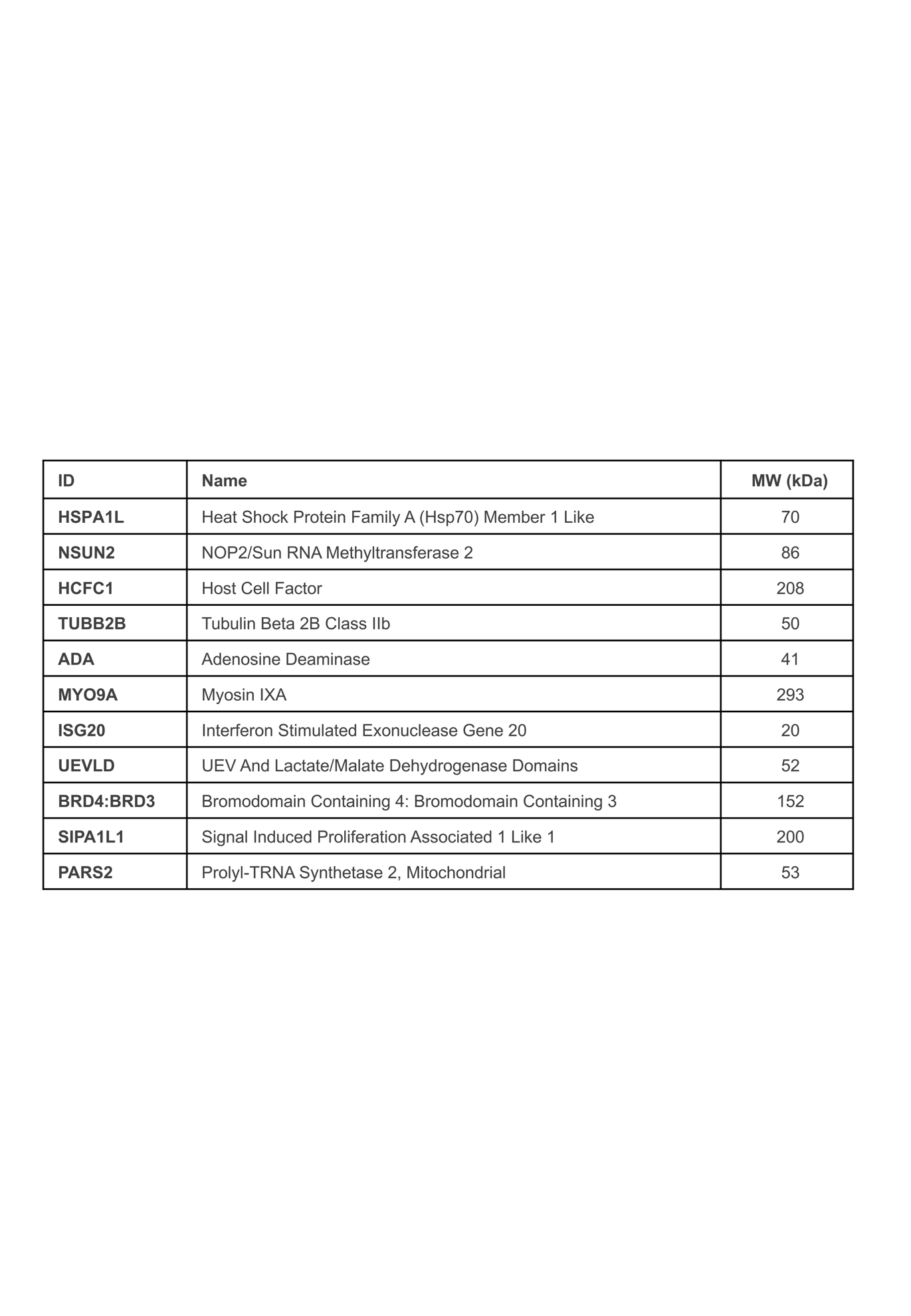


**Data file S1. Interactors PKCα WT, PKCα-D463H, or PKCα-D463N.** Proteins co-immunoprecipitated with MYC-tagged PKCαWT and mutants D463H and D463N from U87MG cells were analysed by label free quantitative mass spectrometry**.** All binding partners pulled down by WT or either D463 mutant, which are not found in any the control immunoprecipitates, are presented as the LFQ intensities; data are also presented as a clustered heat map (see main text Figure 4D).
